# Sample prep for proteomics of breast cancer: proteomics and gene ontology reveal dramatic differences in protein solubilization preferences of radioimmunoprecipitation assay and urea lysis buffers

**DOI:** 10.1186/1477-5956-6-30

**Published:** 2008-10-24

**Authors:** Lambert CM Ngoka

**Affiliations:** 1 Department of Chemistry, Virginia Commonwealth University, 1001 West Main Street, P. O. Box 842006, Richmond, VA 23284-2006, USA; 2 Department of Biomedical Sciences, Paul L. Foster School of Medicine, Texas Tech University Health Sciences Center, MSB-1, 5001 El Paso Drive, El Paso, TX 79905, USA

## Abstract

**Background:**

An important step in the proteomics of solid tumors, including breast cancer, consists of efficiently extracting most of proteins in the tumor specimen. For this purpose, Radio-Immunoprecipitation Assay (RIPA) buffer is widely employed. RIPA buffer's rapid and highly efficient cell lysis and good solubilization of a wide range of proteins is further augmented by its compatibility with protease and phosphatase inhibitors, ability to minimize non-specific protein binding leading to a lower background in immunoprecipitation, and its suitability for protein quantitation.

**Results:**

In this work, the insoluble matter left after RIPA buffer extraction of proteins from breast tumors are subjected to another extraction step, using a urea-based buffer. It is shown that RIPA and urea lysis buffers fractionate breast tissue proteins primarily on the basis of molecular weights. The average molecular weight of proteins that dissolve exclusively in urea buffer is up to 60% higher than in RIPA.

Gene Ontology (GO) and Directed Acyclic Graphs (DAG) are used to map the collective biological and biophysical attributes of the RIPA and urea proteomes. The Cellular Component and Molecular Function annotations reveal protein solubilization preferences of the buffers, especially the compartmentalization and functional distributions.

It is shown that nearly all extracellular matrix proteins (ECM) in the breast tumors and matched normal tissues are found, nearly exclusively, in the urea fraction, while they are mostly insoluble in RIPA buffer. Additionally, it is demonstrated that cytoskeletal and extracellular region proteins are more soluble in urea than in RIPA, whereas for nuclear, cytoplasmic and mitochondrial proteins, RIPA buffer is preferred.

Extracellular matrix proteins are highly implicated in cancer, including their proteinase-mediated degradation and remodelling, tumor development, progression, adhesion and metastasis. Thus, if they are not efficiently extracted by RIPA buffer, important information may be missed in cancer research.

**Conclusion:**

For proteomics of solid tumors, a two-step extraction process is recommended. First, proteins in the tumor specimen should be extracted with RIPA buffer. Second, the RIPA-insoluble material should be extracted with the urea-based buffer employed in this work.

## Background

Over the past few years, proteomics has emerged as a powerful new technology, capable of generating unprecedented details of protein maps in a wide range of cell types and disease processes. Increasingly, however, it is becoming recognized that the success of a proteomic experiment is critically dependent on the sample preparation step. An ideal sample prep protocol should not only isolate as much of the proteins of interest as possible from the biological source, but also preserve optimal sample integrity and morphology. It should also present the entire sample in a form that is compatible with optimum mass spectrometric analysis.

Proteins in their native states are generally embedded in their natural environments where they are associated with other proteins, biological macromolecules or other matrix materials. They may also be components of multi-protein complexes, integrated into plasma membranes or organelles. They are generally insoluble in their native states once isolation from their biological environments. They must therefore be denatured in order to bring them into solution. This ultimately entails dissociating the chemical bonds connecting them in their native states. The bonds, and appropriate agents/methods for dissociating them [1] include: disulfide bond (*reduction & alkylation*), hydrogen bond (*chaotropes*), electrostatic interactions (*salts*, *charged detergents*, *chaotropes*), charge-dipole (*chaotropes*), dipole-dipole (*strong dipolar molecules*), van der Waals (*salt*, *dipolar molecules*, *chaotropes*), and hydrophobic interactions (*salts*, *dipolar molecules*, *chaotropes*).

Effective sample preparation for proteomics disrupts these associations, and solubilizes as large a subset of the proteins as possible. Sample solubilization buffers typically contain a number of additives (chaotropes, detergents, reducing agents, buffers, salts, and ampholytes). In proteomics, perhaps two of the most effective and widely employed lysis buffers for extracting proteins from cells and tissues are **R**adio-**I**mmunoprecipitation **A**ssay **b**uffer (RIPA buffer) [2] and urea lysis buffer [1,3,4].

The base ingredients of a typical RIPA buffer include: 50 mM Tris HCl pH 8, 150 mM NaCl, 1% NP-40, 0.5% sodium deoxycholate and 0.1% SDS. Protease and phosphatase inhibitors are additionally added prior to use, depending on the application (these are usually not added when preparing lysates for phosphatase assays). Additional optimization of the lysis procedure, or substitution of the base ingredients may be required for each specific application, for example PBS pH 7.4 can substitute for both Tris HCl and NaCl. An example variant of RIPA buffer that contains protease and phosphatase inhibitors consists of: 10 mM Tris, pH 7.4, 100 mM NaCl, 1 mM EDTA, 1 mM EGTA, 1 mM NaF, 20 mM Na_4_P_2_O_7_, 2 mM Na_3_VO_4_, 0.1% SDS, 0.5% sodium deoxycholate, 1% Triton-X 100, 10% glycerol, 1 mM PMSF (made from a 0.3 M stock in DMSO) or 1 mM AEBSF (water soluble version of PMSF), 60 μg/mL aprotinin, 10 μg/mL leupeptin, 1 μg/mL pepstatin (alternatively, protease inhibitor cocktail may be used).

RIPA buffer's rapid and efficient cell lysis and solubilization of a wide range of proteins, including cytoplasmic, membrane and nuclear proteins, makes it a standard for Western blotting. Its versatility is further augmented by its compatibility with protease and phosphatase inhibitors, stability, and ability to minimize non-specific protein-binding interactions leading to low backgrounds in immunoprecipitation. Still, RIPA buffer is very compatible with a myriad of applications, including reporter assays, protein assays, immunoassays and protein purification. When protein quantitation is desired, RIPA buffer is the lysis buffer of choice due to its compatibility with the BCA Protein Assay, although it can denature kinases [5], and can disrupt protein-protein interactions in immunoprecipitation/pull down assays [6].

Urea buffer is another versatile and efficient cell and tissue lysing buffer whose typical composition include: TRIS base 40 mM, Urea 7 M, Thiourea 2 M, NP-40 or CHAPS 4%, DTT 10 mM. Urea is used at concentrations ranging from 5 to 9 M, often with thiourea at concentrations up to 2 M. The additive thiourea can dramatically enhance the solubility of a wide range of proteins – nuclear, membrane, cytosolic, and including even tubulin that is highly prone to aggregation, in urea buffer [1,3,4]. As in RIPA buffer, different detergents and buffers can be substituted for the buffering base, NP-40 or CHAPS, depending on the application. Urea inactivates proteases that degrade cellular proteins [7]. Therefore, there is little need to add protease inhibitors. However, urea and thiourea can hydrolyze to cyanate and thiocyanate, respectively, which can modify amino groups on proteins, (e.g. carbamylation of proteins by isocyanate), and this hydrolysis is promoted by heat.

Extracellular matrix (ECM) proteins (collagens, fibronectin, vitronectin, thrombospondin, laminins, tenascin, osteopontin and entatin, etc.) are highly implicated in tumor cell growth, motility, angiogenesis, apoptosis, proliferation, invasion and metastasis [8-22]. They contribute to signal transduction, differentiation, site-specific gene expression, immunological functions, wound healing and inflammation [8,15,17,18,22,23]. They and their remodeling enzymes, including urokinase plasmogen activators, matrix metalloproteinases and cathepsins L, B and D, are also known to profoundly modulate mammary gland branching morphogenesis [24]. They are also important biomarkers of breast cancer. For example, Veeck and co-workers [9] showed recently that the extracellular matrix protein Inter-α-trypsin inhibitor is strongly down-regulated in all human breast cancers.

Thus, in cancer proteomics, especially where it is desired to recover as much of the cellular or tissue proteins as possible, it is important not to completely rely on a single lysis buffer that could exclude an entire class of critically needed proteins, especially the low abundance proteins. Indeed, there are numerous proteomics publications that fall exactly under the above category. In breast cancer proteomics, for example, articles are found (example Refs [23,25-30]) wherein researchers wanted to extract all proteins present in the breast tumor specimens, but used RIPA buffer as the sole buffer.

In a study of ECM components in differentiating teratocarcinoma cells, Grover and Adamson [31] noted that fibronectin was poorly solubilized in RIPA buffer. Otherwise, no other direct reference suggesting insolubility of ECMs in RIPA buffer was found as yet. Instead, researchers (examples: Refs [9,13-15,20,32]), routinely use RIPA buffer as the sole lysis and solubilization buffer in the immunoprecipitation [15], immunoblot [13,14,20] and Western blot [9,32] analysis of extracellular matrix proteins.

## Methods

**Figure 1:**
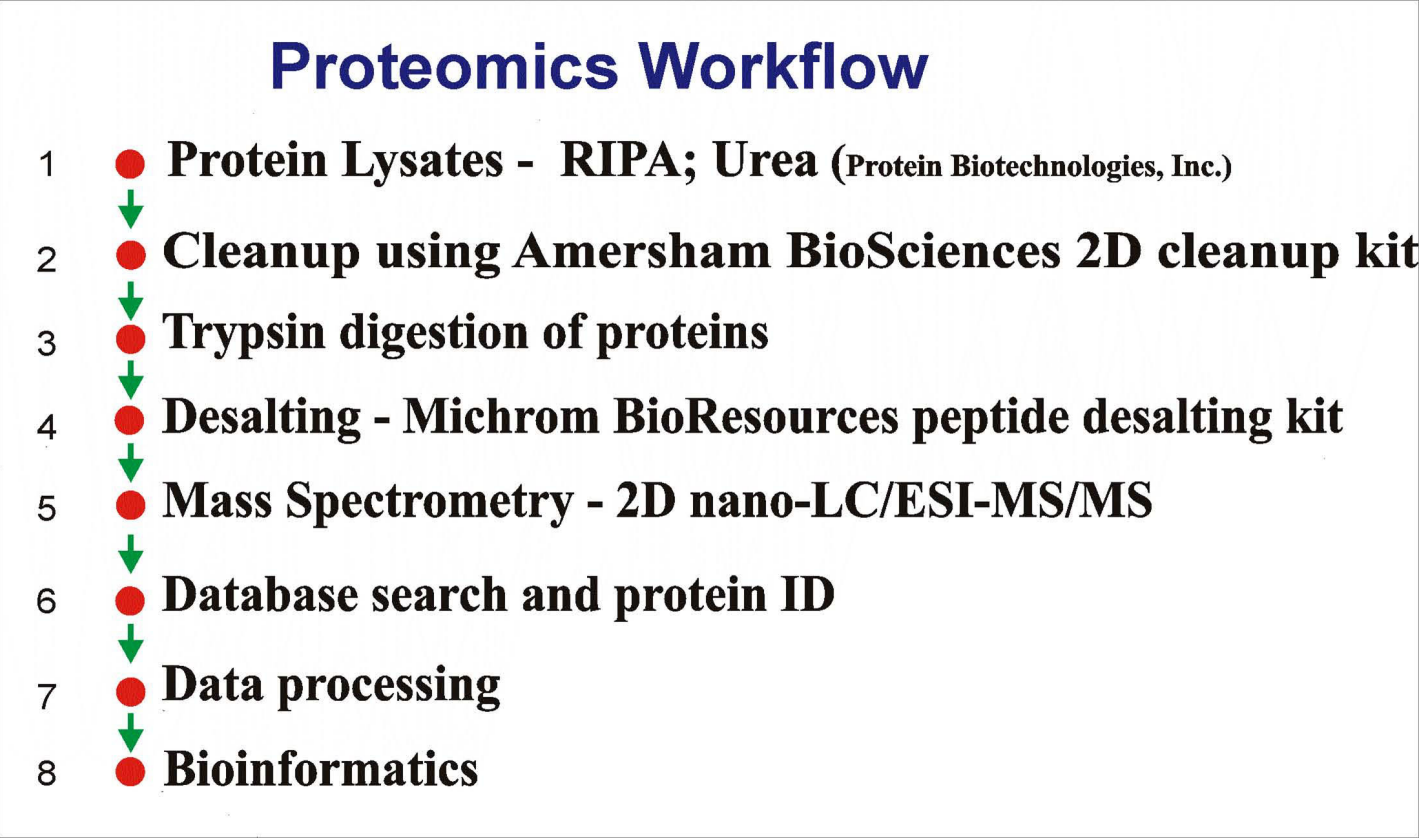
Proteomics Workflow: flow chartoutlining the key experimental steps in this work.

The key steps (Figure 1) include protein extraction from breast tumors and matched normal breast tissues, sample clean-up with GE Healthcare tools, trypsin digestion, desalting with Michrom cartridges, mass spectrometry/2D nano-LC/ESI-MS/MS, database search and protein ID, data processing and bioinformatics.

### Breast tumor tissue lysates

Protein lysates of breast tumors and matching normal breast tissues are obtained from Protein Biotechnologies, Inc. (Ramona, CA). The samples are selected to represent different types of tumors (T2-018 Tumor, T2-048 Tumor, T2-029 Tumor), non-neoplastic normal breast tissue (T2-048 Normal), Diagnosis, Stage, Grade, Age and Gross Findings (Table 1).

**Table 1:** Characteristics of the breast tumors and matched normal breast tissues analyzed in this work.

	**T2-018 Tumor**	**T2-048 Tumor**	**T2-048 Normal**	**T2-029 Tumor**
**Location**	Right breast	Left breast	Left breast	Bilateral

**Diagnosis**	Infiltrating ductal carcinoma	Infiltrating ductal carcinoma	Normal	Adenocarcinoma

**Stage**	IV T4N0M0	IIB T2N1M0	Normal	IV T4N1M0

**Grade**	II	I	I	III

**Sex**	Female	Female	Female	Female

**Age**	75 years	39 years	39 years	47 years

**Gross Findings**	Tumor size: 8 × 8 cm., ill demarcated. Cut section firm	Tumor size: 3 × 3.5 cm. ill demarcated. Cut section soft and white.	Tissue size: 3 × 3.5 cm.	Tumor size 2 × 2 cm., and 3 × 2 cm., ill demarcated. Cut section firm and gray/white

Source: Integrated Laboratory Services-Biotech (ILSbio), Chestertown, MD 21620. .

Detailed histopathology reports are available at the Protein BioTechnologies website :

### Protein extraction

Lysates provided by Protein Biotechnologies were extracted by a two-step procedure. First, proteins are extracted with a modified **R**adio-**I**mmunoprecipitation **A**ssay (RIPA) lysis buffer to yield the soluble fraction. Second, the residual insoluble fraction left after RIPA buffer extraction is subjected to additional extraction using a urea-based buffer to produce a second protein fraction. The compositions of the lysis buffers are as follows:

#### Extraction 1

Radioimmunoprecipitation Assay Lysis Buffer (modified). Base Ingredients (PBS, pH 7.4, SDS, 0.1%, Na-deoxycholate, 0.25%); RIPA Protease Inhibitors (1 mM, Phenylmethylsulfonyl fluoride (PMSF), EDTA, 1 mM (calcium chelator; 100 mM stock solution in H_2_O, pH 7.4), Leupeptin, 1 μg/mL, Aprotinin, 1 μg/mL, Pepstatin, 1 μg/mL, NaF (1 mM),); RIPA Phosphatase Inhibitors (Activated Na_3_VO_4 _(1 mM)).

#### Extraction 2

Urea Lysis Buffer (PBS, pH 7.4, 5.0 M Urea, 2.0 M Thiourea, 50 mM DTT, 0.1% SDS).

### Cleanup of lysates

In the cleanup step, the proteins are separated from buffers, detergents, salts and other contaminants, using a method that is largely derived from a protocol and 2D clean-up kit provided by Amersham Biosciences (GE Healthcare, Piscataway, NJ). The kit consists of four reagents: a precipitant that precipitates the proteins to form pellets, a co-precipitant that co-precipitates with the proteins and enhances their removal from solution, a wash buffer that removes non-protein contaminants from the protein precipitate, and a wash additive that promotes rapid and complete re-suspension of the sample proteins.

Prior to the beginning of clean-up, the wash buffer was chilled at -20°C for 1 hr. After thawing and spinning down 100 μg aliquots of breast tumor lysates and matched normal breast tissue lysates, 300 μL of the precipitant was added. The mixture was vortexed on Eppendorf Thermomixer R (Eppendorf North America, Westbury, NY), and then incubated in ice for 15 minutes. Next, 300 μL of co-precipitant was added and the mixture mixed. The mixture was centrifuged at 12000 × g for 5 minutes to pellet the proteins. The clear supernatant liquid was carefully pipetted out while retaining the protein precipitate at the bottom of the 1.5 mL Eppendorf tube. Without disturbing the pellet layer, 40 μL of co-precipitant was added to the supernatant, through the tilted side of the 1.5 mL Eppendorf tube. The mixture was kept in ice for 5 minutes before centrifuging it again at 12000 × g for another 5 minutes. The pellet was dispersed by adding 25 μL of MilliQ water and centrifuging for 10 minutes. After adding 1 mL of chilled wash buffer at -20°C and 5 μL of wash additive, the mixture was vortexed once every 30 seconds for a total of 35 minutes. At this point, the proteins did not dissolve, but dispersed. The mixture was again centrifuged at 12000 × g for 5 minutes. The supernatant was carefully discarded, and the pellet dried. The pellets are amorphous.

### In-solution digestion

The dried pellet was re-suspended in 20 μL 8 M urea/100 mM ammonium bicarbonate (ABC), and 0.6 μL of 100 mM Dithiothreitol (DTT) in 100 mM ABC (i.e. 3 mM DTT) was stirred in Eppendorf Thermomixer R (Eppendorf North America, Westbury, NY) for 1 hr at 29°C. After adjusting to room temperature, 1.5 μL of 200 mM iodoacetamide (IAA) in 100 mM ABC (final concentration of 15 mM IAA) was added. Alkylation was then carried out by incubating the mixture for 45 minutes in a darkroom. Then, 1.5 μL of 200 mM DTT/100 mM ABC was added to consume any un-reacted IAA. The urea concentration was reduced to about 1 M by diluting the mixture with 140 μL of (50 mM ABC + 2 mM CaCl_2_). Digestion was carried out by adding 6 μL of 0.40 μg/μL = 2.4 μg of Promega Sequencing Grade trypsin and incubating in Eppendorf Thermomixer R for 20 hr at 37.4°C.

At the end of the 20-hr incubation, reaction was stopped by adding 4.0 μL of 2% acetonitrile. Then, 6 μL of 10% TFA was added to adjust the pH to 5.0.

### Desalting

Manual, Micro trap desalting cartridge and protocol from Michrom (Michrom BioResources, Auburn, CA) are used. First, the micro Trap is washed with 80 μL of LCMS Solvent B (90%ACN/0.1% TFA). Next, it is equilibrated with 80 μL of LCMS Solvent A (2% ACN/0.1% TFA). Then, 20 μL of peptide digest sample is loaded onto the micro Trap; salts are removed by washing with 50 μL aliquots of LCMS solvent A (2% ACN/0.1% TFA). Tryptic peptides are eluted from the micro Trap with 16 μL of 70% ACN. Desalted peptides are evaporated to dryness on an SC2 SpeedVAC^® ^Plus Thermo savant (Thermo Fisher Scientific, Waltham, MA).

### Multidimensional nanoHPLC

The nano-HPLC is a Paradigm MS4B Multi-Dimensional HPLC equipped with a Michrom Paradigm AS1 refrigerated autosampler and χCalibur software plugin (Michrom BioResources, Auburn, CA). It is configured and operated in a 3-1 column-switching arrangement. All four pumps are used; pump D is used for sample loading onto captrap cartridge (sample concentration and de-salting) at 50 μL/minute for 5 minutes. Solvents A is 100% MilliQ water, no additives added. Solvent B is HPLC Grade acetonitrile (ACN) from Burdick and Jackson (Honeywell Burdick & Jackson, Morristown, NJ), no additives added. Solvent C is 100 mL of FA/HFBA mix + 900 mL water; (FA = formic acid, HFBA = HPLC Grade Ionate™ Heptafluorobutyric acid from Pierce (Pierce Biotechnologies, Rockford, IL)). The FA/HFBA mix consists of 10% FA + 0.5% HFBA + 89.5% water. Solvent D is made by mixing 10 mL FA/HFBA mix + 20 mL ACN + 970 mL water.

The stock solution used to make up the MudPIT salt solutions is prepared as follows: 5% ACN + 90% water + 2.5% formic acid + 2.5% ammonium hydroxide + 0.05% HFBA. The salt plugs for MudPIT analysis are then prepared by serial dilution of this stock solution with solvent D described above.

An optimized 60-minute nano gradient consists of the following settings:

*Time (min), Flow rate (μl/min), B (%), C (%) *:: (0.00, 0.30, 5.00, 10.00 :: 5.00, 0.30, 5.00, 10.00 :: 12.00, 0.30, 15.00, 10.00 :: 47.00, 0.30, 40.00, 10.00 :: 55.00, 0.30, 60.00, 10.00 :: 56.00, 0.30, 80.00, 10.00 :: 58.00, 0.30, 80.00, 10.00 :: 59.00, 0.30, 5.00, 10.00 :: 65.00, 0.30, 5.00, 10.00).

The five Solvent System and Event Group 2(D2) pulse sequences for autosampler, mass spec and solvent flow as follows: *Time (min), Event *:: (0.00, Valve 1 inject :: 0.05, Valve 2 inject :: 5.00, Valve 2 Load :: 5.10, Start Mass Spec :: 0.00, Valve 1 inject), whereas three Solvent System and Event Group 1(D1) settings for sample concentration and desalting on trap column are: *Time (min), flow (μL/min) *:: (0.00, 50 :: 5.00, 50 :: 5.10, 5.00).

### Columns (Michrom BioResources)

• Peptide Nanotrap (TR5/25109/42): 150 μm × 50 mm; 400 nL volume.

• Nanotrap analytical column (CL5/61241/00): 5 μm 200 Ǻ Magic C_18 _75 μm × 150 mm.

• SCX Captrap (TR1/25108/35): Contains a medium pore, large particle, silica-based strong cation exchange material (PolySulfoethyl Aspartamide). Binds protein digests, peptides, and other molecules (0.5–50 kD) for 1D or 2D analysis. Concentrates samples up to 100 fold (pH range 2.7–7.0).

### Nano-LC/MS/MS

One-dimensional (1D) and two-dimensional (2D) nano HPLC experiments are run at a flow rate of 300 nL/min. Samples are loaded onto trap columns for concentration and desalting at 50 μL/min. For each experiment, 12 μL of peptide digest resulting from 50–100 μg total protein is used: 2 μL is injected for 1D; 10 μL for 2D. Each shotgun experiment consists of a 12-cycle MudPIT run in which a 60-minute nano-LC gradient is run for each of: 1D, 2D, 2D (0 mM NH4COO), 2D (25 mM NH4COO), 2D (50 mM NH4COO), 2D (75 mM NH4COO), 2D (100 mM NH4COO), 2D (150 mM NH4COO), 2D (200 mM NH4COO), 2D (250 mM NH4COO), 2D (300 mM NH4COO) and 2D (500 mM NH4COO).

### Nanospray

The nanospray is a Paradigm Nanotrap Platform (Michrom BioResources, Auburn, CA) equipped with a Paradigm Metal spray needle. The spray tip is a 7.5 cm long, 30 μm (Internal Diameter) × 105μm (Outer Diameter) surgical stainless steel, electrochemically cut and polished, and sheathed by a 125 μm PEEK Tubing. The needle permits flow range of 0.5 to 10 μL/min and a voltage range of 1000 to 5000 Volts. A 1/16" stainless steel Valco nut attaches the spray needle to a 1/16" to 1/16" Valco union, which is mounted on the Nanotrap Platform.

### Nanospray source parameters

Sheath Gas Flow Rate = 0; Aux Gas Flow Rate = 0; Spray Voltage (kC) = 2.51; Spray Current (μA) = -0.05; Capillary Temp (°C) = 221.10; Capillary Voltage (V) = 9.22; Tube Lens Offset (V) = 50.

### Mass spectrometry

Data-dependent MS and MS/MS spectra are acquired on an LCQ Deca Xp plus (Thermo Fisher Scientific, San Jose, CA).

### MS and MS/MS

Five scan events are recorded for each data acquisition cycle. The first scan event is used for full scan MS acquisition from 300–1800 *m/z*. Data are recorded in the **centroid **mode only (scan event #1 does not permit **profile **mode of data acquisition). The remaining four scan events are used for Collisionally Activated Decomposition (CAD): the four most abundant ions in each MS are selected and fragmented to produce product ion mass spectra. All CAD product ions are recorded in the **profile **mode.

### Ion optics settings used

Multipole 1 Offset (V) = -6.15; Lens voltage (V) = -24.23; Multipole 2 Offset (V) = -10.54; Multipole RF Amplitude (V_p-p_): = 400; Entrance Lens (V) = -50.79.

### MS/MS parameters

Number of microscans: 4; Maximum Injection Time (ms): 200; Isolation width (*m/z*): 3; Normalized Collision Energy (%): 35; Activation Q: 0.250; Activation Time (msec): 30.00; Scan Range: 300–1800 *m/z*.

### Database searches and protein identification

Proteins are identified by searching the MS and MS/MS spectra against NCBI nr human fasta, using Bioworks v3.2 (Thermo Fisher Scientific, Inc., San Jose, CA). The Unified Search Results File format (.SRF) is employed, rather than the traditional SEQUEST *.DTA and *.OUT formats. Peptide and protein hits are scored and ranked using the new probability-based scoring algorithm and the new final Score (Sf) that are incorporated in Bioworks 3.2. Because MS/MS spectra are acquired in the profile mode, each nano-LC/MS/MS run range in size from 95–120 Megabytes, depending on the number of microscans (typically 4 ms) in the Advanced Define Scan function in the MS/MS **LCQtune **file of the Instrument Method.

### Filters

Only peptides identified as possessing fully tryptic termini (containing up to two missed internal trypsin cleavage sites), with cross-correlation scores (*X*_corr_) greater than 1.9 for singly charged peptides, 2.3 for doubly charged peptides and 3.75 for triply charged peptides, are used for peptide identification. In addition, the delta-correlation scores (ΔC_n_) must be greater than 0.1 for peptide identification. Protein probability P(pro) ≤ 0.001.

### Bioinformatics

Bioinformatics calculations are carried out using Blast2GO [33] Version 1.2.7 , a java webstart-enabled Gene Ontology annotation, visualization and analysis software. The calculations, as implemented here, consist of three key sequential steps: (a) Basic Local Alignment Search Tool (BLAST) [34,35], (b) Mapping and (c) Annotation.

### BLAST

In BLAST [34], protein input queries are submitted to the BLAST server at the National Center for Biotechnology Information (NCBI) of the National Institutes of Health (NIH) over the internet . The BLAST server generates hits (hit gene ids (gi) and gene names/accessions). The input parameters used are as follows: BLAST database, NCBI nr; number of BLAST hits requested for each query, 40; BLAST expectValue (*i.e. *eValue), 1e^-3^; BLAST program, blastp; Blast Versions: BLASTP 2.2.13 [Nov-27-2005] and BLASTP 2.2.15 [Oct-15-2006]; Blast Mode: QBlast-NCBI; HSP length cutoff: 33 (please see "***The NCBI Handbook"***[36] for more information on BLAST parameters).

The blast server accepts only ***fasta***-formatted protein sequences as input queries. Although Bioworks 3.2 or later can convert protein sequences into ***fasta ***text files, the protein sequences must be submitted from within Bioworks browser prior to exiting the initial protein identification step. Thus, batch conversion of protein queries, post-Bioworks, is not possible *via *Bioworks route. For example, Bioworks would not allow the analysis of only the proteins that occur in both tumor and normal, because these must be determined post-Bioworks protein identification. Another approach is **Batch Entrez **at the NCBI website ), with *Protein database* selected to import the batch file and displaying all in ***fasta ***format.

For each of the protein input queries, the **BLAST **machine generates a **BLAST Table **[34], exemplified here (Additional File 1) with discoidin domain receptor 1 (DDR1): gi|68533097|dbj|BAE06103.1|/894 (DDR1 is found in T2-081-T1, T2-018T2, T2-029-T2, T2-048N1, and T2-048N2 (Table 1)). The **BLAST Table **shows parameters of the BLAST search: Sequences producing significant alignments, Gene Name, Accession number, e-Value, align-length, positives, similarity %, hsp and mapping (Ontologies found), for each of the 40 hits requested.

### Mapping

In the Mapping step, various databases are searched to identify and fetch Gene Ontologies (GO) associated with the hits obtained from NCBI BLAST searches.

### Annotation

The annotation procedure selects the GO terms from the GO pool obtained by the mapping step and assigning them to the query sequences, using Annotation Rule. Annotation parameters are: Pre-eValue-Hit-Filter, 6; Pre-Similarity-Hit-Filter, 30; Annotation Cut-Off, 55; GO-Weight, 5.

Annotations are validated and expanded using an annotation expander. The expander, developed by a group at the Norwegian University of Science and Technology , deploys an additional Gene Ontology structure: the Second Gene Ontology Layer, to suggest new Biological Processes and Cellular Components, based on the gene's existing Molecular Function annotations.

## Results

The multidimensional protein identification technology (MudPIT) mass spectra of the breast specimens **T2-018 Tumor, T2-048 Tumor, T2-048 Normal **and **T2-029 Tumor**, are shown in Additional Files 2, 3, 4 and 5, respectively. The set of 12-cycle spectra to the left of the figures are the RIPA buffer fractions, whereas the spectra of the 12 urea buffer fractions are shown at right.

The mass spectra clearly show that, for each of the 60-minute MudPIT runs, the 1D_2 μL and 2D_10 μL spectra appear to produce higher ion currents than the rest of the spectra. This may be partly due to the greater concentrations of peptides in these runs: in the 1D_2 μL run, all two 2 μL of approximately 5 μg total peptide digest are introduced into the nano-LC/ESI-MS/MS system via the peptide Nanotrap and analytical column (the SCX column is bypassed for 1D analysis). And, in 2D_10 μL, all 10 μL of peptide digest are deposited on the SCX column; all unbound peptides are washed into the mass spectrometer, after pre-concentration and de-salting at the 150 μm × 50 mm peptide (40 nanoliter volume) nanotrap. Thus, the amounts of sample introduced into the mass spectrometer in these two runs maybe responsible for the higher ion currents.

The MudPIT spectra also show that the RIPA-insoluble materials contain quite a significant amount of proteins, as reflected in the highly abundant spectra of urea-soluble proteins. This clearly raises concern about using RIPA buffer as the sole lysis buffer for the proteomics of breast cancer (or other cancers as well).

The proteins identified in the database search of the MudPIT mass spectra are shown in Table 2. Here, **T2-048T (RIPA) and T2-048N2 (UREA) **represent the RIPA-soluble fraction of the tumor sample **T2-048 **and the urea-soluble fraction of the normal breast tissue sample **T2-048**, respectively. The column labelled "**Default**" depicts the default number of proteins found by Bioworks 3.2 prior to application of filter functions and protein validation. The final sets of proteins identified are shown at the rightmost column. Again, it is clear that the RIPA-insoluble materials, which dissolve in urea, contain significant amounts of proteins that would otherwise be discarded if RIPA buffer was the only lysis and solubilization buffer used.

**Table 2:** Proteins found in the MudPIT proteomics of the RIPA and urea fractions of the breast tumors analyzed in this work.

			**# *Proteins***	**# *Proteins_validated***
	**Lysate**		**Default**	**• *Xcorr *(1,2,3): 1.90, 2.3, 3.75** **• *P(pro) *: 0.001** ** • Δ*Cn *≥ 0.1**
		
***Specimen***	**RIPA Buffer**	**Urea Buffer**		

T2-018_Tumor	*		33147	299

T2-018_Tumor		*	31828	155

T2-048_Tumor	*		27049	143

T2-048_Tumor		*	31329	261

T2-048_Tumor	*		28203	195

T2-048_Tumor		*	30816	122

T2-029_Tumor	*		27944	215

T2-029_Tumor		*	24963	358

**Figure 2:**
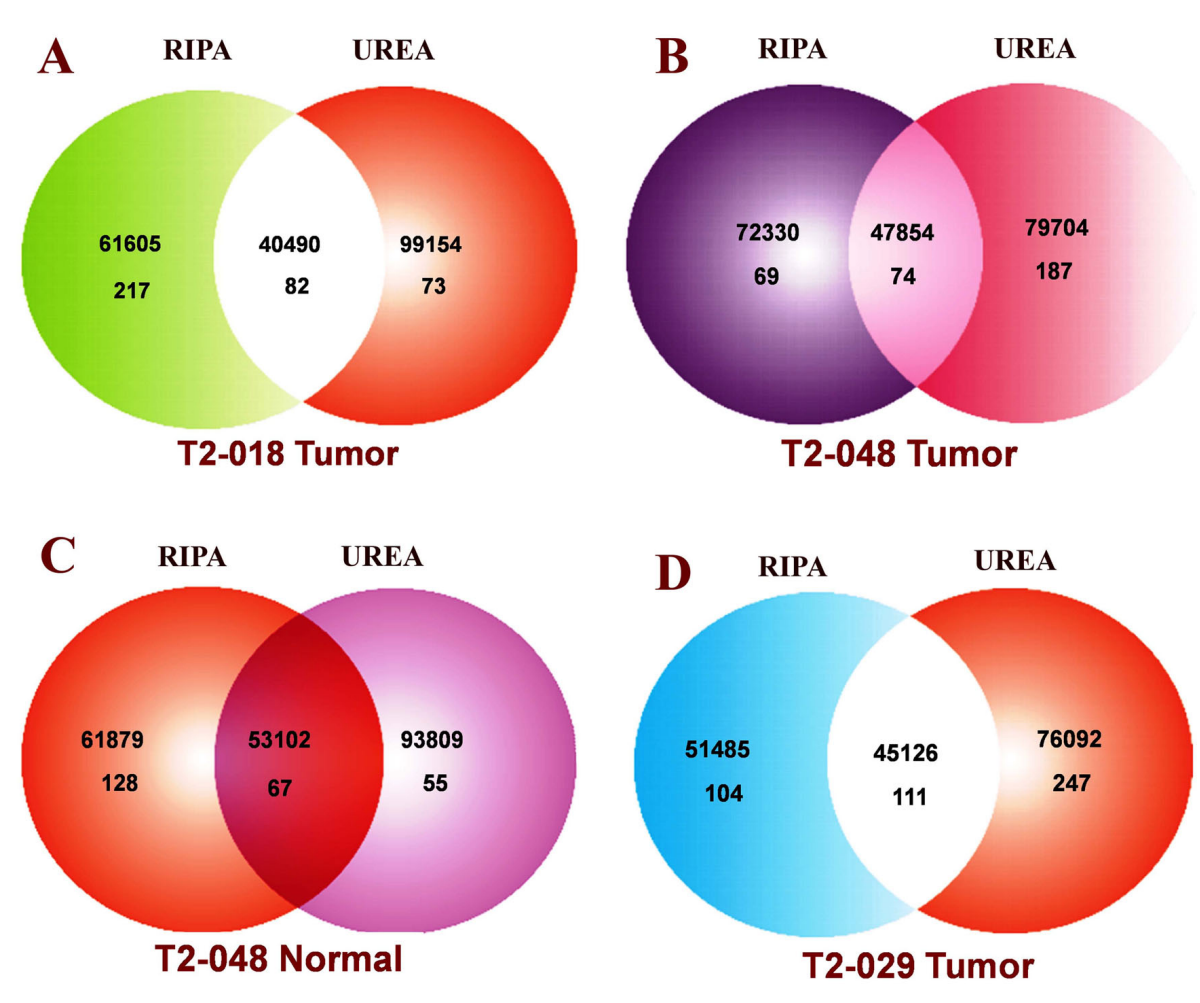
**(A-D): A: The partitioning of the proteins between RIPA and urea buffers in the infiltrating Ductal Carcinoma case T2-018T (TUMOR)**. Venn diagram showing that RIPA and urea lysis buffers fractionate breast tumor proteins primarily on the basis of molecular weights. The average molecular weight of the 217 proteins that dissolve exclusively in RIPA buffer is 61604 *m/z*, whereas the 73 proteins that dissolve exclusively in urea buffer have an average molecular weight of 99154 *m/z*. B: Differential partitioning of proteins extracted from the T2-048 TUMOR between RIPA and urea buffers. Venn diagram showing that RIPA and urea lysis buffers fractionate breast tumor proteins on the basis of molecular weights. The average molecular weight of the 69 proteins that dissolve almost exclusively in RIPA buffer is 72330 *m/z*, whereas the 187 proteins that dissolve exclusively in urea buffer have an average molecular weight of 79704 *m/z*. Seventy-four proteins, whose average molecular weight is 47854*m/z*, dissolve fully in both RIPA and urea buffers. C: Matched normal breast tissue T2-048 NORMAL between RIPA and UREA buffers. The average molecular weight of the 128 proteins that are soluble nearly exclusively in RIPA buffer is 61879 *m/z*, whereas the 55 proteins that dissolve exclusively in urea buffer have an average molecular weight of 93810 *m/z*. Sixty-seven proteins with an average molecular weight of 53102*m/z *are fully soluble in both RIPA and urea buffers. D: Bilateral Adenocarcinoma case T2-029 TUMOR. The average molecular weight of the 104 proteins that dissolve almost exclusively in RIPA buffer is 51485 *m/z*, whereas the 247 proteins that dissolve exclusively in urea buffer have an average molecular weight of 76092 *m/z*. One hundred and eleven proteins with an average molecular weight of 45126 *m/z *are fully soluble in both RIPA and urea buffers.

The partitioning of the proteins between RIPA and urea buffers in the infiltrating Ductal Carcinoma case **T2-018 TUMOR **(Figure 2A) shows that the average molecular weight of the 217 proteins that dissolve exclusively in RIPA buffer was 61604 *m/z*, whereas the 73 proteins that dissolve exclusively in urea buffer have an average molecular weight of 99154 *m/z*. That is, the average molecular weight of proteins that dissolve exclusively in urea was 37551 *m/z*, or 61% higher than in RIPA buffer. Finally, the average molecular weight of the 82 proteins that dissolve in both RIPA and urea buffers was 40490 *m/z*.

The corresponding data for T2-048 TUMOR, T2-048 NORMAL and T2-029 TUMOR are shown in Venn Diagrams in Figures 2B, C and 2D.

### Gene Ontology

In an effort to determine the protein groups, and identify the fractionated proteins contained in the compartments shown in Table 2 and Figure 2, Gene Ontology [37,38] was employed.

The Gene Ontology (GO) project, which began in 1998 as a collaboration between three databases **FlyBase, *Saccharomyces *Genome Database **and the **Mouse Genome Database**, has today grown to encompass nearly all major databases, and has become a new powerful tool for mining biology data. Gene Ontology annotates biological data in terms of their **Biological Process, Molecular Function **and **Cellular Component**.

**Biological Process **is an ensemble of biochemical transformations that are accomplished by one or more ordered assemblies of molecular functions. Biological Process may be broad (physiological process, metabolism, etc) or specific (nitric oxide metabolism, oxygen transport, etc).

**Molecular Function **is the specific, elemental action or task performed by a gene product or assembled complexes of gene products. Examples of broad molecular functions include catalysis, binding, and structural molecular activity, whereas specific molecular functions are exemplified by tetrapyrrole binding, adenylate cyclase activity and calmodulin binding.

**Cellular Component **is the subcellular location (organelle, nucleus, etc) and macromolecular complexes were the gene product is located.

In this work, proteins identified by proteomic analyses are submitted to GO analyses, including NCBI BLAST, mapping and annotation. The results are presented as a **Directed Acyclic Graph **(DAG), which shows the number of annotated sequences and the annotation scores contributing to each node. The nodes are color-coded, and the relative importance of each annotation score is indicated by the intensity of the orange color at that node. There are three types of nodes: a double-edged octagon represents an annotated GO term; a rectangle represents a non-annotated GO term node, and an oval shape denotes Gene Ontology obtained by mapping which can directly be directly associated to one or more BLAST hits.

In the Biological Process formulation, functionalities that directly identify the proteins in the RIPA and urea buffer fractions are not explicitly evident. The Molecular Function annotations, and especially, the Cellular Component, however, do provide highly useful information on protein groups and identities of the protein fractions, shown in the Venn diagrams of Figure 2. In the following sections, the Cellular Component annotations will be primarily used to characterize the protein fractions, although, the Molecular Functions annotations will also be used.

### Cellular Component

#### T2-018T (RIPA) and T2-018T (Urea) fractions

**Figure 3:**
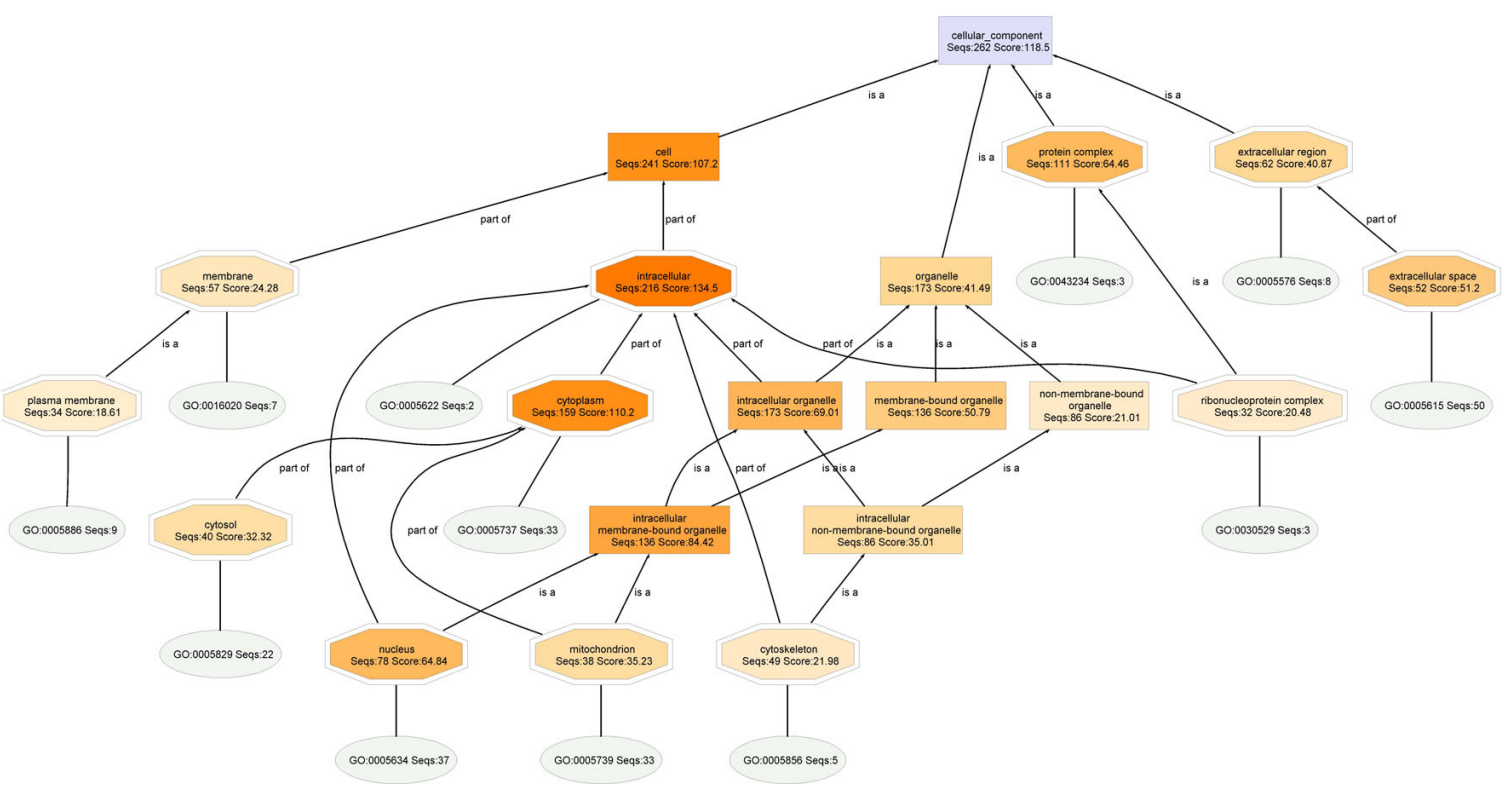
**The Cellular Component DAG for the proteome T2-018T (RIPA)**. Extracellular matrix proteins are not observed, even at a node filter setting of 28.

**Figure 4:**
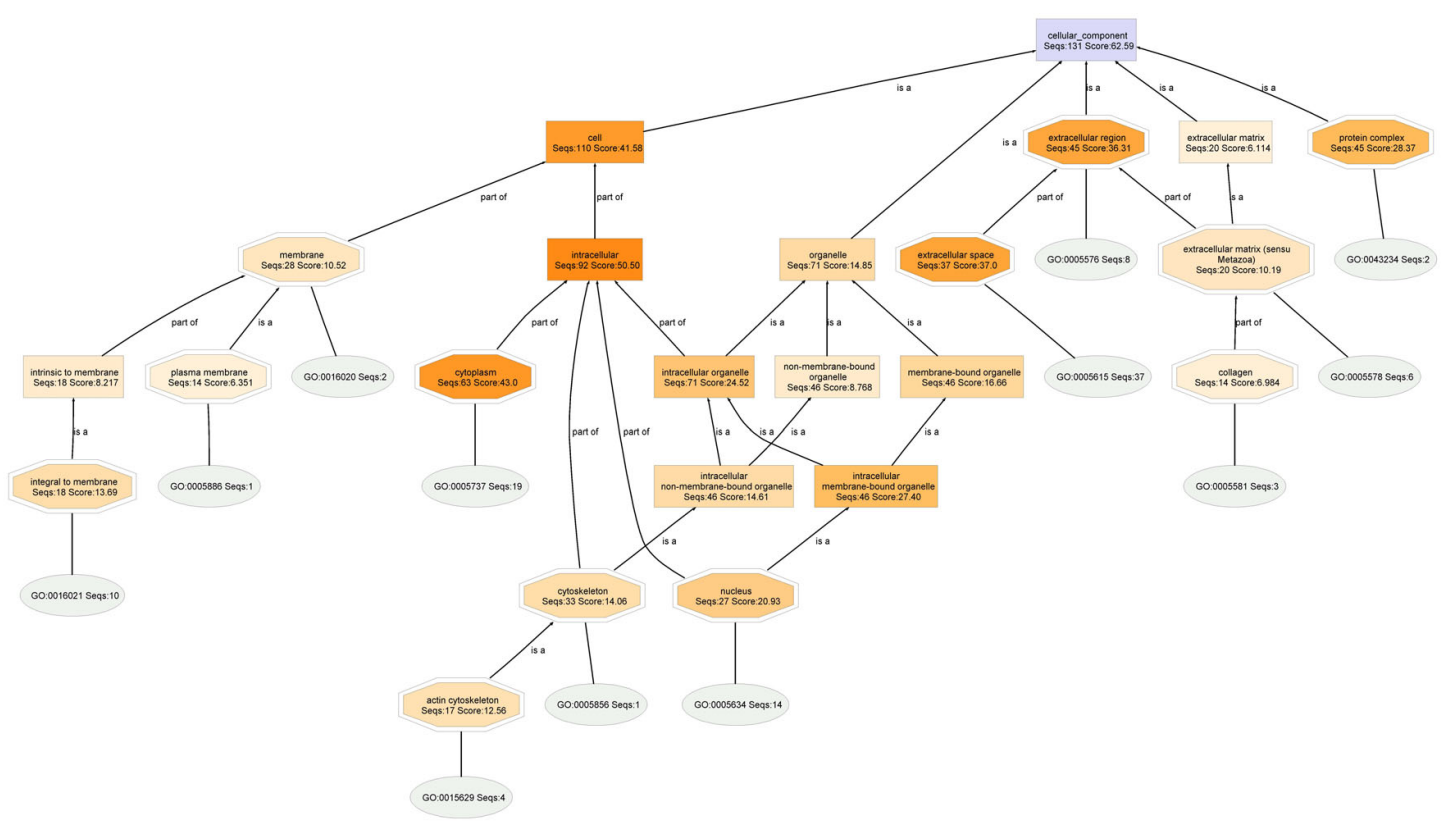
**The Cellular Component DAG for the proteome T2-018T (UREA)**. At a node filter setting of only 13, extracellular matrix proteins are highly evident. Thus, extracellular matrix proteins are soluble primarily in urea buffer.

The Cellular Component DAGs for specimens **T2-018T (RIPA) **and **T2-018T (Urea) **are shown in Figures 3 and 4, respectively. Interestingly, the Cellular Component parent node of **T2-018T **DAG shows that mapping found curated Gene Ontologies (Ontologies) for 262 out of the original 299 proteins found in this sample (Table 2). Similarly, in **T2-018T**, 131 out of the 155 proteins have Ontologies available in the Go databases. The high percentages of proteins that have curated Ontologies thus provide adequate bioinformatics data needed to characterize the RIPA and urea proteomes with high degree of specificity and reliability.

Comparison of the **T2-018T (RIPA) **and **T2-018T (Urea) **DAGs shows that the entire set of extracellular matrix protein nodes in the urea (**T2-018T**; Figure 4) DAG are almost completely missing from the RIPA (**T2-018T**; Figure 3) DAG, at the indicated node filter settings, suggesting that nearly *all extracellular matrix proteins are dissolved in the urea*, but not in the RIPA buffer.

The node filter is a mechanism of simplifying otherwise complicated DAGs. At a node filter of 28, for example, all nodes whose number of annotated protein sequences are 28 or below, are not displayed, n an effort to simplify the DAG. Thus, when the node filter is lowered, previously hidden nodes are displayed, making the chart more crowded.

**Figure 5:**
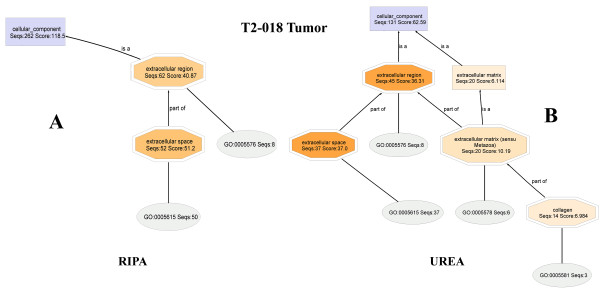
**Close-up views of the extracellular regions of T2-018T (UREA)**. clearly show that extracellular matrix proteins dissolve almost exclusively in urea buffer.

**Close-up **sections of the extracellular regions (Figure 5) clearly show that extracellular matrix proteins dissolve almost exclusively in urea buffer. The cropped sections are obtained with node filters of 15 and 10, for the RIPA and urea DAGs, respectively.

**Figure 6:**
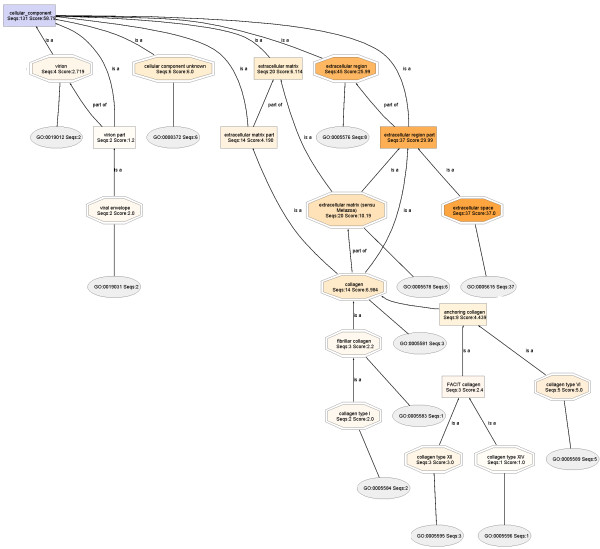
**Expanded view of the extracellular region of the cellular component DAG for the proteome T2-018T (UREA)**. The node filter was reduced to 0 to obtain this complete display. Lowering the DAG node filter for the RIPA DAG did not produce appreciable change in the number of nodes displayed within the extracellular region.

Interestingly, lowering the DAG node filter for the RIPA DAG did not produce appreciable change in the number of nodes displayed within the extracellular region shown in Figure 3, whereas, even a slight lowering of the node filter in the urea DAG (Figure 4) reveals a large number of previously hidden nodes, shown here in Figure 6, when the urea node filter is reduced to zero. Again, this is a further confirmation that most of the extracellular matrix proteins are dissolved in the urea buffer.

Comparison also shows that, for extracellular region, RIPA buffer has a greater number of annotated protein sequences and annotation score than Urea buffer: [RIPA: (extracellular region, Seqs:62 Score:40.87) :: UREA: (extracellular region, Seqs:45 Score:36.31)]. However, this greater enrichment at the RIPA buffer node may indeed be due to the greater number of proteins at the RIPA buffer Cellular Component parent node: [RIPA buffer: (Cellular Component, Seqs:262 Score:118.5) :: UREA buffer: (Cellular Component, Seqs:131 Score:62.59)]. To correct for this, and allow for sequence-independent comparison of the nodes, regardless of the number of protein sequences that was submitted to GO analyses (Table 2), the sequences and scores are expressed as percentages of the sequences and scores present at the Cellular Component parent node. This process is known as normalization. Thus, comparison of the extracellular regions become: [RIPA: (extracellular region, (Seqs:23.7 Score:34.5)) :: UREA: (extracellular region, (Seqs:34.4 Score:58.0))]. It can now be stated that, based on Seqs, the extracellular region is preferentially enriched in Urea buffer by 34.4%, when compared to RIPA buffer (23.7%). Similar calculations are shown below, for **Seqs **of (**RIPA**/**UREA**), respectively: nuclear (29.8/20.6), membrane (21.8/21.4), intracellular (82.4/70.2), protein complex (42.4/34.4), organelle (66.0/54.2), cytoskeleton (18.7/25.2) and cytoplasmic proteins (60.7/48.1). From here, it is clear that cytoskeletal and extracellular region proteins are selectively enriched in urea buffer, whereas nuclear, intracellular, protein complexes, organelle, cytoplasmic and mitochondrial proteins are preferentially enriched in the RIPA fractions.

**Figure 7:**
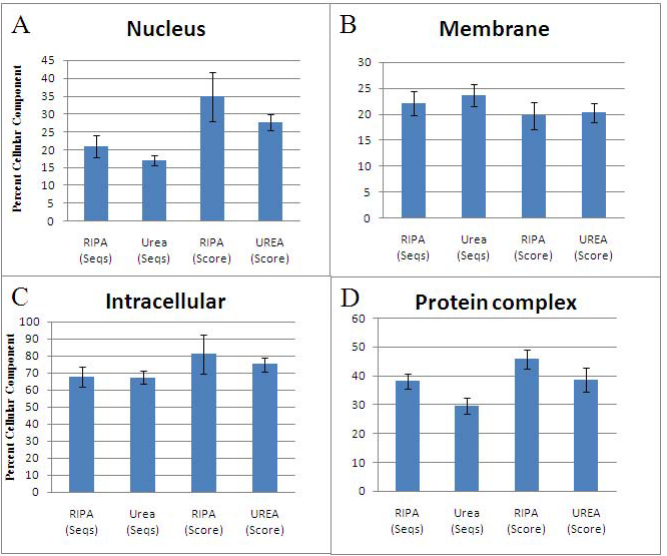
**Extraction of proteins from breast tumors for proteomic analysis**. nuclear proteins (A), intracellular proteins (C) and protein complexes (D), are more soluble in RIPA buffer than in urea buffer RIPA, whereas membrane proteins (B) are slightly more soluble in urea buffer.

**Figure 8:**
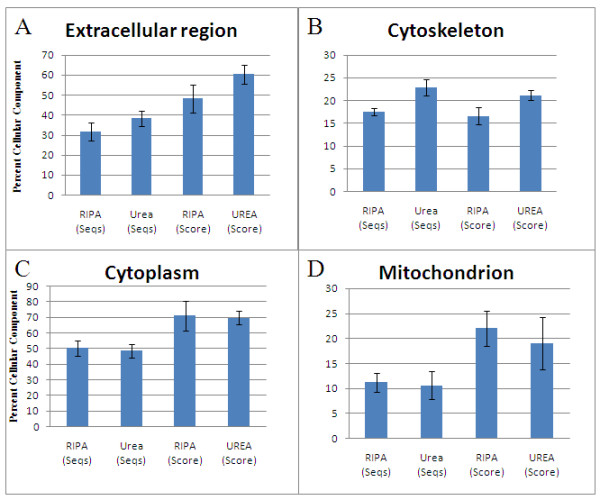
**Extraction of proteins from breast tumors for proteomics**. proteins of the extracellular region (A) and cytoskeleton (B) are more soluble in urea buffer than in RIPA, whereas for cytoplasmic (C) and mitochondrial (D) proteins, RIPA buffer is preferred.

Selective protein enrichment comparisons based on both Seqs and Scores are shown in Figures 7 and 8.

#### T2-048T (RIPA) and T2-048T (Urea) fractions

**Figure 9:**
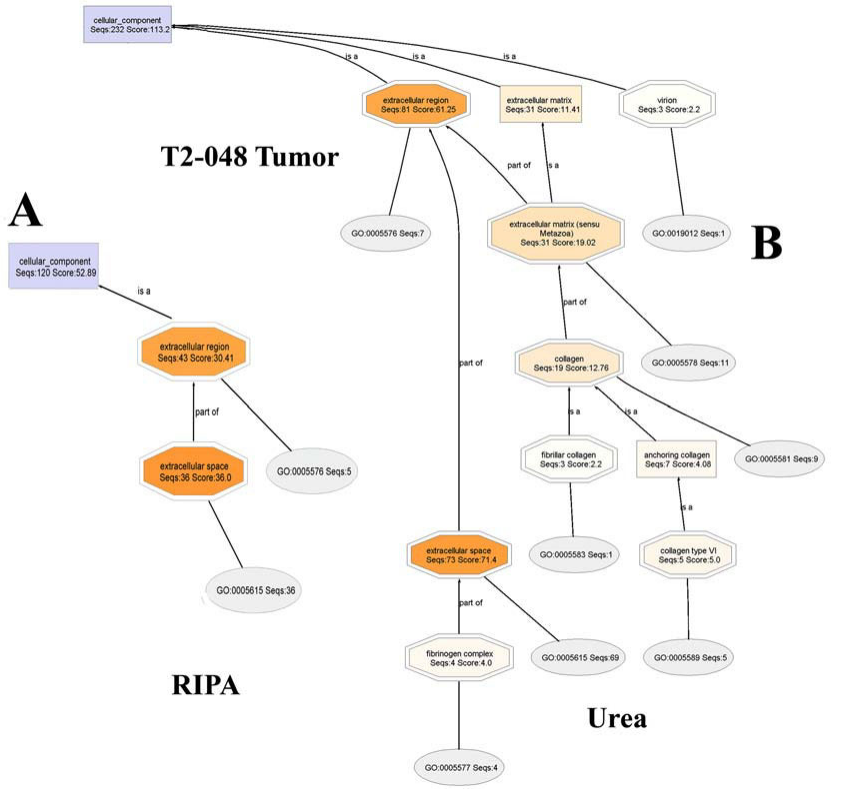
**Close-up views of the extracellular regions of the Cellular Component DAG of the proteomes T2-048T (RIPA) and T2-048T (UREA)**. Extracellular matrix proteins and virions are observed almost entirely in urea fraction.

Rather than show the entire DAGs, cropped views of the extracellular regions of **T2-048T (RIPA) and T2-048T (Urea) **DAGs (Figure 9) show that extracellular matrix proteins are present almost exclusively in the urea fraction. It is also seen that mapping found that nearly 90% of the original proteins present in the **T2-048T **proteome (i.e. 232 of 261, Table 2) have existing Ontologies, thus providing the requisite bioinformatics information needed for the characterization of the proteins. Similarly, Ontologies are found for 120 of the 143 proteins (84%) of the **T2-048T **proteome.

#### T2-048N1 (RIPA) and T2-048N2 (Urea) fractions

**Figure 10:**
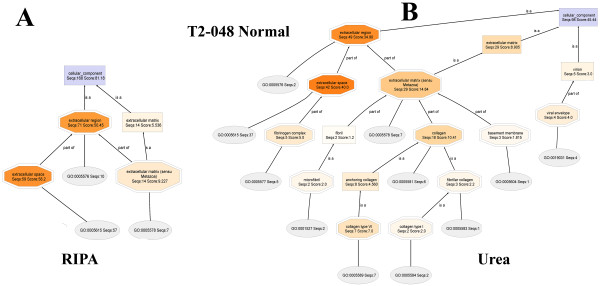
**Close-up views of the extracellular regions of the Cellular Component DAG of the proteomes T2-048N (RIPA) and T2-048N (UREA)**. Extracellular matrix proteins and virions are observed almost exclusively in urea fraction.

Here again, the extracellular regions of **T2-048N1 (RIPA) and T2-048N2 (Urea) DAGs **(Figure 10) show that extracellular matrix proteins are present nearly exclusively in urea buffer, with 86% and 80% of the proteins of **T2-048N1 **and **T2-048N2 proteomes**, respectively, being found to have existing Ontologies available. It is interesting to compare the normalized extracellular matrix Seqs and scores of **T2-048T (Tumor) **and **T2-048N2 (Normal): **[**T2-048T-UREA**: (extracellular matrix (Seqs:13.36 Score:16.80)) :: **T2-048N2-UREA**: (extracellular matrix, (Seqs:29.59 Score:32.66))]. The normalized scores and Seqs belonging to normal breast tissues are more than two times those of the breast tumors, which may be due to the degradation of extracellular matrix proteins in the tumor by proteases to pave the way for the metastasis of cancer cells [8-22].

#### T2-029T1 (RIPA) and T2-029T2 (Urea) fractions

**Figure 11:**
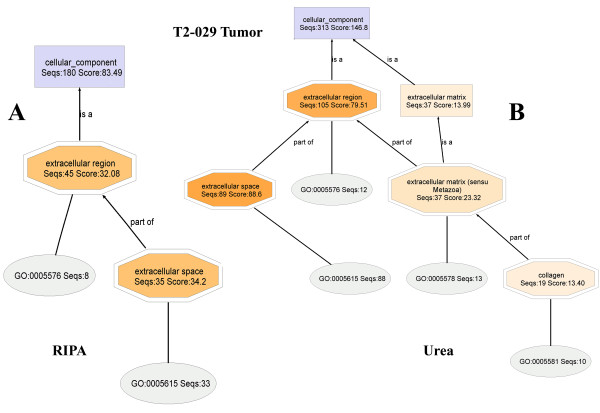
**Close-up views of the extracellular regions of the Cellular Component DAG of the proteomes T2-029T (RIPA) and T2-029T (UREA)**. Extracellular matrix proteins are observed almost exclusivly in urea fraction (right).

The cropped-out extracellular regions, displayed side-by-side (Figure 11), again show that extracellular matrix proteins are found nearly exclusively in urea buffer. And, mapping found Ontologies for 84% and 87% of the **T2-029T1 **and **T2-029T2 **proteins, respectively. Upon lowering the node filter on the **T2-029T2 **DAG to zero, a full display of the extracellular matrix proteins are obtained (Additional File 6). Similar lowering of the node filter on the **T2-029T1 **DAG did not reveal significantly new or relevant structural information.

Complete Cellular Component DAGs for **T2-048T (RIPA) and T2-048T (Urea), T2-048N1 (RIPA) and T2-048N2 (Urea)**, and **T2-029T1 (RIPA) and T2-029T2 (Urea)**, are available as Additional Files 7, 8, 9, 10, 11 and 12, respectively.

### Molecular Function

Molecular Function annotations also contain elements that reveal protein solubilization preferences of the RIPA and urea buffers. Thus far, Structural Molecule Activity (SMA) is the only functionality in the Molecular Function annotation that contains nodes that directly relate to the protein solubility preferences. In general, the SMA node of the urea proteome contains a child node, **Extracellular Matrix Structural Constituent**, which contains the number of annotated protein sequences and an annotation score for **extracellular matrix proteins**. The SMA node of the RIPA proteome does not contain any descriptors (at the given node filter settings) that suggest the presence of **Extracellular Matrix Structural Constituent**. Thus, extracellular matrix proteins are observed nearly exclusively in the urea fraction.

**Figure 12:**
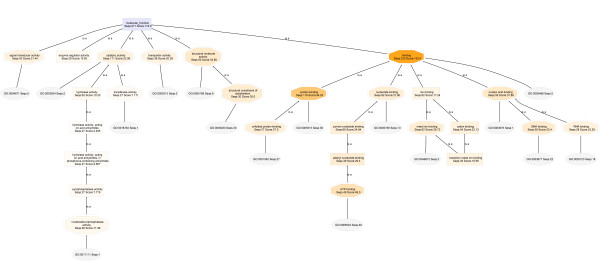
**Molecular Function DAG for the proteome T2-018T (RIPA)**. Extracellular matrix structural constituents are not seen, even at a node filter setting of 12.

**Figure 13:**
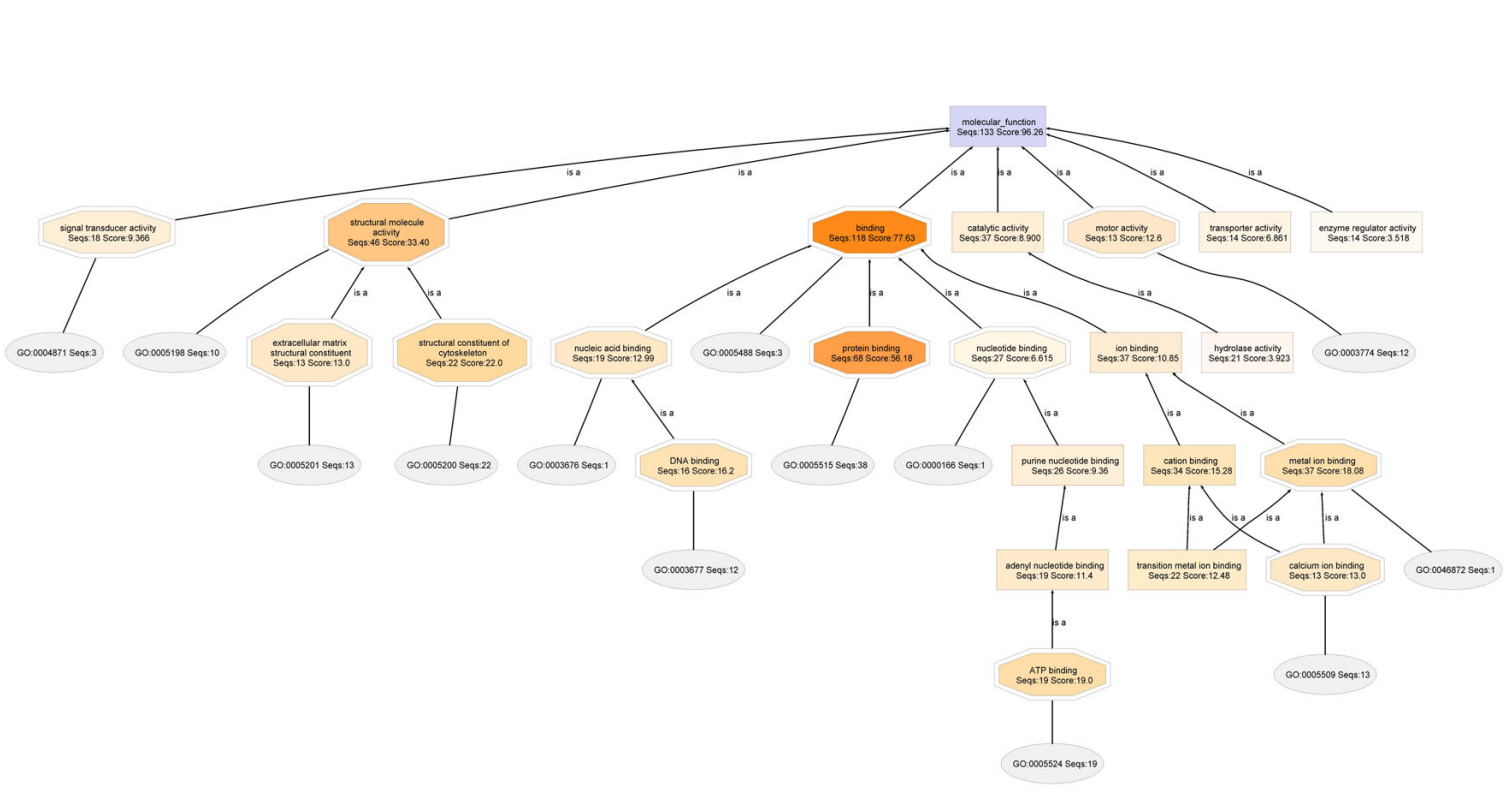
**Molecular Function DAG for the proteome T2-018T (UREA)**. The Structural Molecule Activity of the urea proteome contains 13 extracellular matrix structural constituents. None is observed in the RIPA buffer fraction DAG of Figure 12 shown above. Thus, extracellular matrix proteins are soluble primarily in urea buffer.

The Molecular Function DAGs for **T2-018T (RIPA) **and **T2-018T (UREA) **(Figures 12 and 13, respectively) show clearly that **Extracellular Matrix Structural Constituents **are present only in the urea proteome. The RIPA and urea DAGs are drawn with node filter settings of 30 and 12, respectively.

**Figure 14:**
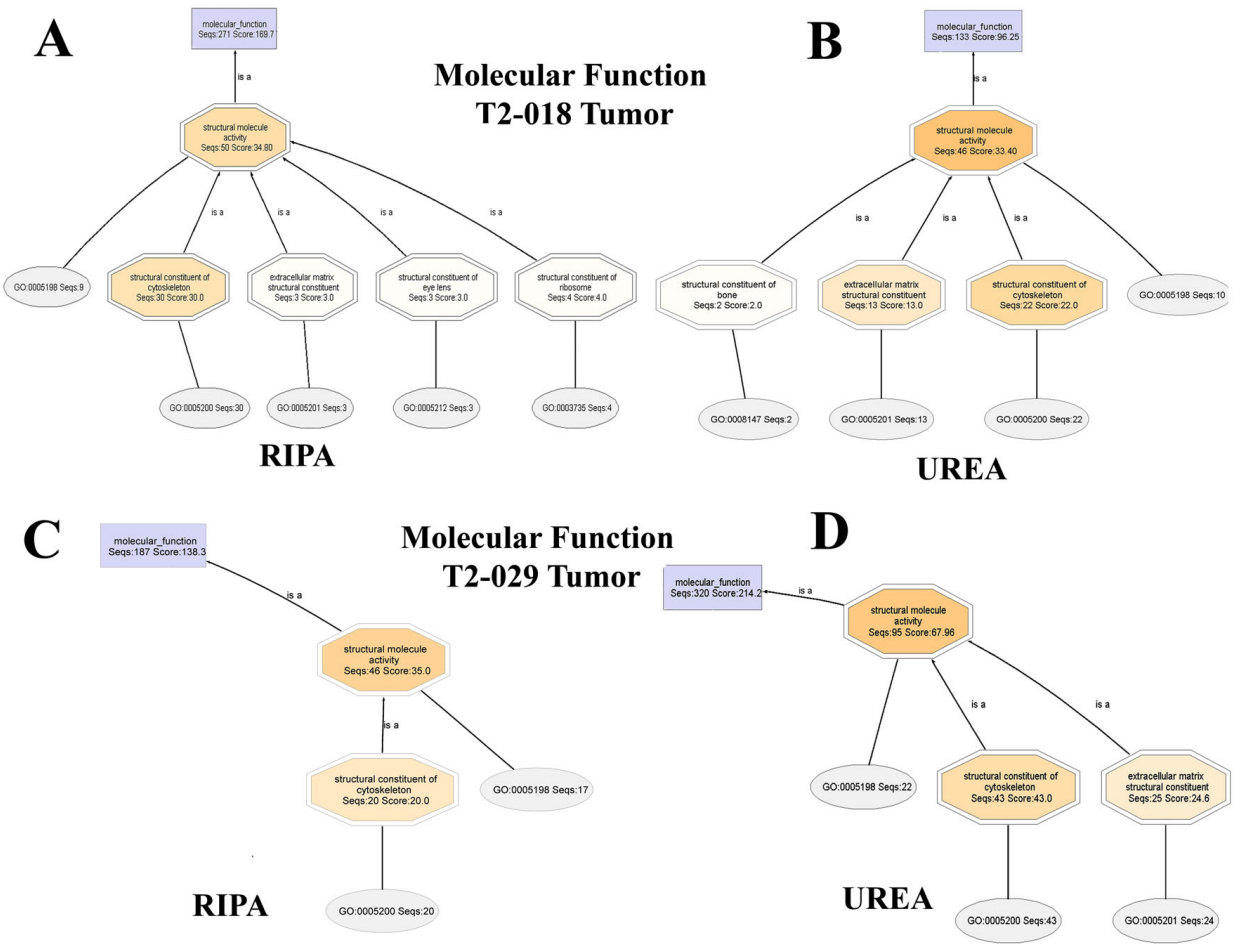
**Close-up views of the Structural Molecule Activity node of T2-018T (RIPA), Panel A, compared to the SMA of T2-018T (UREA), Panel B**. The SMA of T2-018T (UREA) contains a node corresponding to extracellular matrix structural constituents, whereas no ECM constituents are seen in the SMA of T2-018T (RIPA). Panels C and D compare the SMA nodes of T2-029T (RIPA) and T2-029T (UREA), respectively, wherein it is seen that no extracellular matrix structural constituents are present in T2-029T (RIPA).

In Figure 14(A–B) are the cropped-out SMA nodes for **T2-018T (RIPA) **and **T2-018T (UREA)**, when the node filters are set to 2 and 1, respectively. Further lowering of the node filters did not produce significant differences in both DAGs. Also shown in Figure 14(C–D), are the cropped-out SMA nodes for **T2-029T1 (RIPA) **and **T2-029T2 (UREA) **proteomes. Again, extracellular matrix proteins are found nearly exclusively in the urea fractions.

**Figure 15:**
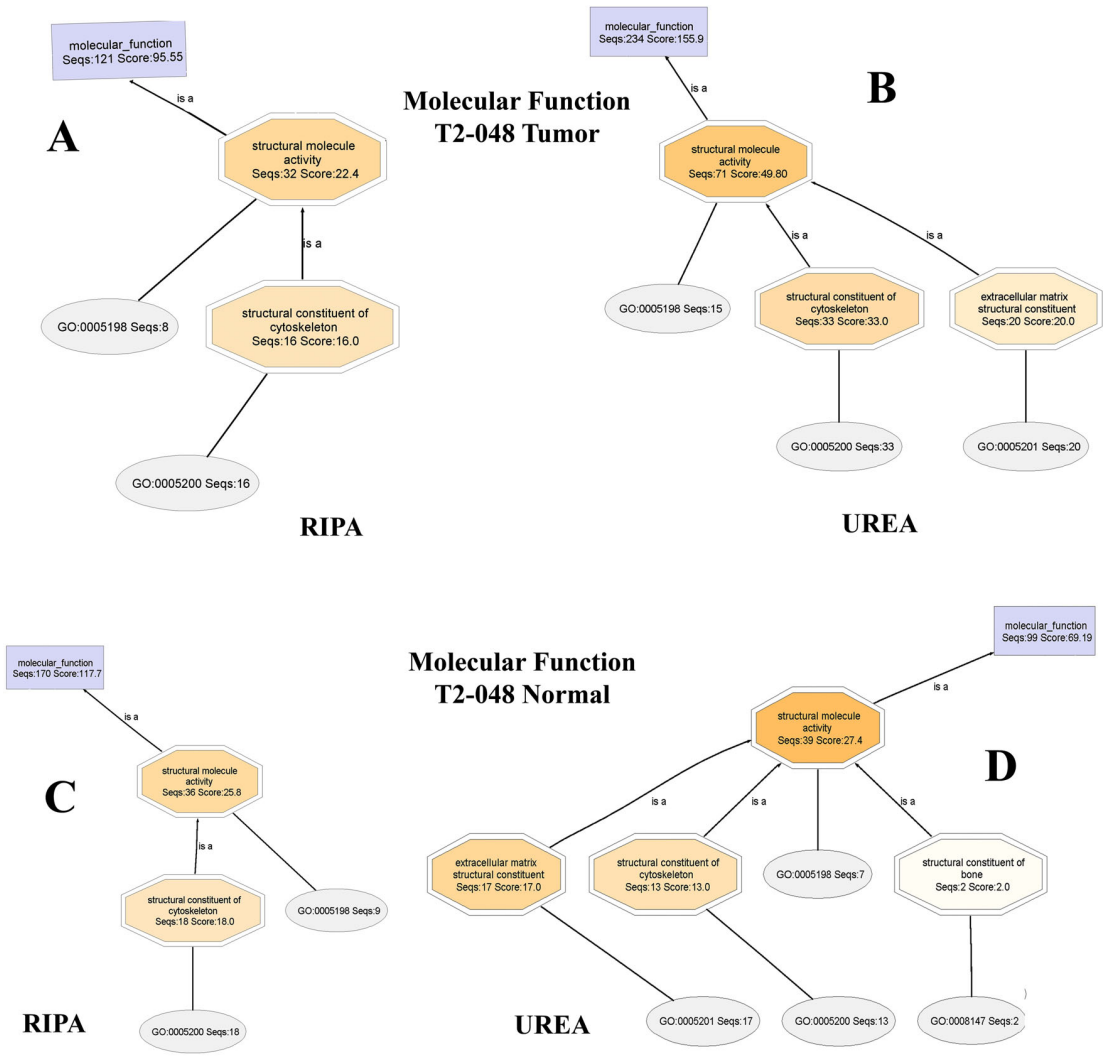
**Close-up views of the Structural Molecule Activity of the Molecular Function DAG of the proteomes T2-048T (RIPA) and T2-048T (UREA), and T2-048N (RIPA) and T2-048N (UREA)**. Extracellular matrix structural constituents are observed nearly exclusively in the urea fractions, Panels B and D.

The above trend is consistently maintained in Figure 15 (**A-D**) for **T2-048T (RIPA) **and **T2-048T (UREA)**, and **T2-048N1 (RIPA) **and **T2-048N2 (UREA)**, respectively.

Detailed Molecular Function DAGs for **T2-048T (RIPA) and T2-048T (Urea), T2-048N1 (RIPA) and T2-048N2 (Urea)**, and **T2-029T1 (RIPA) and T2-029T2 (Urea)**, are available as Additional Files 13, 14, 15, 16, 17 and 18, respectively.

## Discussion

The solubility of proteins in RIPA and urea buffers depends on several physicochemical factors, including the characteristics of the proteins and properties of the RIPA and urea buffers.

Physicochemical properties of the proteins that affect their solubility include average charge, determined by the relative numbers of Asp, Glu, Lys, and Arg residues, and the content of turn-forming residues (Asn, Gly, Pro, and Ser) [39]. Insoluble proteins tend to have more hydrophobic stretches longer than 20 amino acids residues, lower glutamine content, fewer negatively charged residues, and higher percentages of aromatic amino acid residues than soluble ones [40]. Indeed, high contents of negatively charged amino-acid residues and absence of hydrophobic patches tend to improve protein solubility [41]. Also, low percentage of aspartic acid, glutamic acid, asparagines and glutamine residues increases the probability of a protein to be insoluble [41].

Solubility of proteins in lysis buffer also depends highly on the composition and gross physicochemical properties of the lysis buffer. These include [42,43]: the type of buffer, the presence or absence of phosphate, pH, salts, ampholytes, detergents, chaotropic agents, reducing agents (dithiothreitol (DTT), dithioerythreitol (DTE), β-mercaptoethanol, tributyl phosphine (TBP), tris-carboxylethylphosphate (TCEP)).

### RIPA and UREA buffers fractionate breast cancer proteins primarily on the basis of molecular weights

RIPA buffer is a versatile and efficient lysis buffer suitable for the recovery of most proteins, including whole cell, nuclear, mitochondrial, membrane receptors, cytoskeletal-associated, and soluble proteins. However, as the data in Figures 2 shows, there is a high molecular weight cut-off (≥ 12% higher average molecular weight in urea than RIPA) for a protein's solubility in RIPA buffer. Proteins with molecular weights of around 100 kDa or higher may not dissolve readily, unless they possess unique structural features that enhance their solubility in RIPA buffer. Thus, when a given protein group is subjected to RIPA buffer extraction, the high molecular weight fraction may not dissolve – they are recovered as the RIPA-insoluble fraction that ultimately dissolve in urea buffer. This may explain why such a high percentage of proteins are recovered in the urea fraction after they have resisted solubility in RIPA buffer (Figures 2D, especially Figures 2D (**T2-029 (TUMOR**)).

Interestingly, Ignatoski and co-workers [42] have also demonstrated that different lysis buffers solubilized different subsets of cellular proteins (rather than entire proteins), based primarily on the molecular weights. Neither RIPA nor urea buffer was, however, used in this kinase assay – they denature kinases. All buffers that they tested were non-denaturing.

### Nearly all extracellular matrix proteins are insoluble in RIPA buffer, but dissolve readily in urea buffer

Perhaps the most important finding in this work is that nearly all extracellular matrix proteins (ECMs) are insoluble in RIPA buffer, whereas they dissolve readily in urea buffer. This may be due to ECMs having very high molecular weights. Why, then, is RIPA buffer being used routinely to dissolve extracellular matrix proteins by researchers, especially in cancer research? Indeed, RIPA buffer does dissolve high molecular weight proteins, but the recovery may be poor. The solubility of a protein is a combination of many factors beyond the nature of the lysis buffer. If, for example, the high molecular weight protein has structural features that enhance its solubility (high contents of negatively charged amino-acid residues and absence of hydrophobic patches [41]), as discussed in the introduction section, the protein would dissolve in RIPA buffer. On the other, a smaller molecular weight protein may surprisingly fail to dissolve in RIPA buffer, if it aggregates or possesses structural features that hamper its solubility. Some epigenetic, post-translation, or spontaneous structural changes can also impede a protein's solubility in RIPA buffer. One example is tau, which would normally be soluble in RIPA. But when it becomes hyperphosphorylated, for example, by endogenously overproduced Aβ protein in Alzheimer's disease, it would resist solubility in RIPA buffer [44].

### Selective Enrichments of Protein Groups by RIPA and Urea Buffers

Data in Figure 7A shows that nuclear proteins are somewhat more selectively enriched in RIPA buffer than in urea, consistent with many standard molecular biology laboratory practices: RIPA buffer is one of the recommended (or one of the preferred) buffers for efficient recovery of nuclear proteins [6,45,46].

Protein complexes (Figure 7D) are also slightly more concentrated in RIPA buffer than in urea. Protein complexes tend to have high molecular weights, and although RIPA buffer has poor solubility for high molecular weight proteins, protein complexes are held together largely by non-covalent bonds. Detailed Cellular Component DAGs (DAGs not shown) indicate that protein complexes referred to here include: immunoglobulin complex, hemoglobin complex, fibrinogen complex, transcriptor factor complex, DNA polymerase complex, DNA-directed RNA polymerase II holoenzyme, RNA polymerase complex, nucleosome, myosin, laminin complex, membrane attack complex, mediator complex, tubulin, MHC protein complex and ribonucleocomplex.

Mitochondrial proteins also appear to be slightly more favored by RIPA buffer than urea (Figure 8D). Again, RIPA buffer is one of the recommended lysis buffers for the recovery of mitochondrial proteins for Western blot [6].

On the other hand, urea buffer is clearly more efficient in selectively enriching extracellular region and cytoskeletal proteins (Figures 7A and 7B), in addition to extracellular matrix proteins already discussed. Neither RIPA buffer nor urea buffer is significantly preponderant in selective enrichment of membrane, intracellular, or cytoplasmic proteins (Figures 7B and 7C, and Figure 8C, respectively).

### Limitations of RIPA and Urea Buffers

There is no single lysis buffer that would solubilize all classes of proteins, however. Each buffer has its pros and cons. Some of the known limitations of RIPA and urea buffers are highlighted below.

### RIPA buffer-induced post-lysis modulation of biochemical pathways

RIPA buffer has been shown to alter some biochemical pathways, leading to experimental results that may be spurious. Hence, the need to verify data by using other lysis buffers. Two examples are provided herein.

In one example, DeSeau and co-workers [47] showed that the level of pp60^c-src ^kinase activity detected in immune complex protein kinase assays can be substantially modulated by RIPA buffer. They, thus, advise that comparing of the results of pp60^c-src ^in vitro protein kinase assays in other cellular systems where only RIPA buffer lysis has been used should be interpreted with caution. Specifically, they found that the *in vitro *protein kinase activity of pp60^c-src ^molecules derived from RIPA buffer lysates of colon carcinoma cells was elevated five- to sevenfold when compared with pp60^c-src ^from the same cells lysed in a buffer containing only Nonidet-P 40. Additionally, they found that in RIPA buffer, the difference in specific activity of pp60^c-src ^between normal colon mucosal cells and colon carcinoma cells is about ten-to thirtyfold, whereas with a lysis buffer containing only Nonidet-P 40 as a detergent, the difference would be less than three- to fourfold. Thus, if Nonidet-P 40 or other lysis buffers were not used in an effort to validate data obtained in RIPA buffer, the entire data on this work could have been in error.

In another example, abnormally high **caspase-3 **and -**7 **activity in stimulated human peripheral blood lymphocytes (PBLs) has been shown to be a spurious side effect caused by RIPA buffer that was used to lyse the activated T-lymphocytes [48]. In contrast, when a lysis buffer containing 2% SDS was used, the **caspase**s remained in their zymogen proforms, and no proteolytic processing of **caspase **substrates was detected. It was subsequently determined that the release liberation of GraB or similar proteases from cytotoxic granules during the lysis procedure was responsible for artifactual activation of **caspase**-3. RIPA may disrupt GraB-containing granules more efficiently than 0.2% Nonidet P-40 or other lysis buffers used [48].

### Many protein groups are insoluble in urea buffer

Although urea buffer has proven very effective in dissolving extracellular matrix proteins and a wide range of other protein groups, it nevertheless has limitations. In fact, Granier [49] noted that many membrane proteins are insoluble in urea, if extracted without heating. And, as mentioned in the Background section above, heating urea in the presence of proteins most likely would result in covalent modifications of the protein by the hydrolysis products produced by heating urea (e.g. carbamylation of proteins by isocyanate). Thus, urea does not possess universal solubility for all membrane proteins. Ames and Nikaido [50] solubilized membrane proteins of *salmonella typhimurium *with hot SDS when even the most powerful O'Farrell's buffer (urea buffer) [51] failed to dissolve the membrane proteins.

A cell surface proteoglycan, with a molecular weight of 450 kDa, was also found to be very insoluble in urea buffer [16].

In general, many proteins that have proven insoluble in urea buffer are shown to be human lens proteins [52,53], especially cataractous proteins [52-54]. Weber and McFadden described a heterogenous set of urea-insoluble proteins in dividing PC12 pheochromocytoma cells. They found that about 5% of the total cellular proteins synthesized in exponentially dividing PC12 pheochromocytoma cells remained insoluble even in 6 M urea [55].

A major factor that decreases the solubility of proteins in urea is the formation of disulfide cross-bridges, which can be acquired by a protein through aerobic oxidation of thiol groups. Even a small molecular weight protein could become very insoluble in urea upon formation of disulphide bridges. This was the case with a 42 kDa Rec12 (Spo11) meiotic recombinase of fission yeast (Rec12 protein) [56] that was expressed in *E. coli*. Rec12 protein resisted solubility in 6 M urea, but was ultimately extracted with 6 M Guanidine hydrochloride [56]. Subsequent analyses showed that it has four disulfide bridges that impeded its solubility in urea. Human eye lens proteins acquire disulfide cross-bridges by exposure to hyperbaric oxygen (Reviewed in Ref. [52]). The eye lens proteins then become opaque, cataractous and resist solubility in urea buffer [52].

Another example is the human centrosomal protein which exists as a doublet of 62/64 kDa and is insoluble in even 8 M urea (a condition that would dissolve most known centrosomal proteins) [57].

### Preferential solubilization of extracellular matrix proteins in urea lysis buffer: variables

Despite differences in the breast tumors analyzed in this work (Table 1), a common feature remains the preferential solubilization of extracellular matrix proteins in urea lysis buffer. Differences include (Table 1): breast location (right breast, left breast, left breast, bilateral); Diagnosis (Infiltrating Ductal Carcinoma, Infiltrating Ductal Carcinoma, Adenocarcinoma), Stage of the tumor (IV T4N0M0, IIB T2N1M0, IIB T4N1M0), Grade of the tumor (II, I, I, III), Patient Age (yrs: 75, 39, 39, 47), and gross findings).

## Conclusion

This work shows that most extracellular matrix proteins (ECM) in the breast tumors and matched normal tissues in this work are dissolved in the urea buffer fraction: they are mostly insoluble in RIPA buffer. Because ECMs are highly important in cancer, including tumor development, progression, adhesion and metastasis, important information may be missed in cancer research if they are not efficiently extracted by RIPA buffer.

This work also shows that RIPA and urea lysis buffers fractionate tissue proteins primarily on the basis of molecular weights. The average molecular weight of proteins that dissolve exclusively in urea buffer is higher (up to 60%) than in RIPA.

Protein complexes, nuclear, mitochondrial, cytoplasmic and intracellular proteins are more soluble in RIPA buffer than in urea, whereas membrane, cytoskeletal and extracellular region proteins are more soluble in urea buffer.

For proteomic analyses of breast tumors, and other solid tumors, a two-step extraction process is herein recommended. First, the tumor should be subjected to RIPA protein extraction. Second, the insoluble matter left after RIPA extraction should be probed for residual protein content by extracting with the urea-based buffer described in this work.

## Competing interests

The authors declare that they have no competing interests.

## Authors' contributions

LCN carried out all of this work, using breast tumor and normal breast tissue lysates provide by Protein Biotechnologies, Inc. (Ramona, VA). Mr. Phillip E. Schwartz, President and CEO of Protein Biotechnologies, Inc. is acknowledged for his generous gift of extra free breast lysates, after an initial purchase from his company. All authors read and approved the final manuscript.

## Supplementary Material

Additional file 1 **BLAST Table for discoidin domain receptor 1 (DDR1).** BLAST Table for discoidin domain receptor 1 (gi|68533097|dbj|BAE06103.1|/894). For each protein input query, the BLASTmachine generates a BLAST Table [34]. The table shows parameters of the BLAST search, including * Sequences producing significant alignments*, * Gene Name*, * Accession number*, * e-Value*, * align-length*, * positives*, * similarity %*, * hsp *and * mapping *(Ontologies found), for each of the 40 hits requested. Click here for file

Additional file 2 **MudPIT Mass Spectra of the breast tumor T2-018 TUMOR.** The set of 12 MudPIT mass spectra of the RIPA-soluble fraction are shown at left, whereas those for the urea-soluble fraction are shown at right. A typical MudPIT experiment consists of a 12-cycle run in which a 60-minute nano-LC gradient is run for each of: 1. 1D_2 μL sample; 2. 2D_10 μL sample; 3. 2D_0 mM NH_4_COO^-^; 4. 2D_25 mM NH_4_COO^-^; 5. 2D_50 mM NH_4_COO^-^; 6. 2D_75 mM NH_4_COO^-^; 7. 2D_100 mM NH_4_COO^-^; 8. 2D_150 mM NH_4_COO^-^; 9. 2D_200 mM NH_4_COO^-^; 10. 2D_250 mM NH_4_COO^-^; 11. 2D_300 mM NH_4_COO^-^, and 12. 2D_500 mM NH_4_COO^-^. NH_4_COO^- ^is ammonium formate. Click here for file

Additional file 3 **MudPIT Mass Spectra of the breast tumor T2-048 TUMOR.** MudPIT Mass Spectra of the breast tumor T2-048 TUMOR. Spectra of RIPA-soluble fraction are shown at left, whereas those for the urea-soluble fraction are shown at right. Click here for file

Additional file 4 **MudPIT Mass Spectra of the matched normal breast tissue T2-048 NORMAL.** The set of 12 MudPIT mass spectra of the RIPA-soluble fraction are shown at left, whereas those for the urea-soluble fraction are shown at right. Click here for file

Additional file 5 **MudPIT Mass Spectra of the bilateral breast tumor T2-029 TUMOR.** The set of 12 MudPIT mass spectra of the RIPA-soluble fraction are shown at left, whereas those for the urea-soluble fraction are shown at right. Click here for file

Additional file 6 **Expanded view of the extracellular region of the Cellular Component DAG for the bilateral proteome T2-029T (UREA).** The node filter was reduced to 0 to obtain this complete display. In contrast, lowering the DAG node filter for the RIPA DAG counterpart did not produce appreciable change in the number of nodes displayed within the extracellular region. Click here for file

Additional file 7 **Cellular Component DAG for the proteome T2-048T (RIPA).** Extracellular matrix proteins are not seen, even when the node filter is lowered to 10. Click here for file

Additional file 8 **Cellular Component DAG for the proteome T2-048T (UREA).** At a node filter setting of 16, there are 31 extracellular matrix proteins. Thus, extracellular matrix proteins are soluble almost exclusively in urea buffer. None is seen in the RIPA buffer fraction of this proteome (Additional File 7). Click here for file

Additional file 9 **Cellular Component DAG of the matched normal proteome T2-048N (RIPA).** Extracellular matrix proteins are not seen, even at a node filter setting of 14. Click here for file

Additional file 10 **Cellular Component DAG of the matched normal proteome T2-048N (UREA).** Twenty-nine extracellular matrix proteins are present in this urea buffer fraction of T2-048N, even when the DAG is displayed with a node filter setting of just 5 (*cf. *Node filter is 14 in Additional File 9 above); no extracellular matrix proteins are present in the RIPA buffer fraction of this proteome, shown in Additional File 9 above. Click here for file

Additional file 11 **Cellular Component DAG of the bilateral Adenocarcinoma proteome T2-029T (RIPA).** Extracellular matrix proteins are not seen, even at a node filter setting of 12. Click here for file

Additional file 12 **Cellular Component DAG of the bilateral Adenocarcinoma proteome T2-029T (UREA).** Thirty-seven extracellular matrix proteins are observed, at a node filter setting of 16. No extracellular matrix proteins are observed in the RIPA buffer fraction of this proteome, which is shown in Additional File 11 above. Click here for file

Additional file 13 **Molecular Function DAG for the proteome T2-048T (RIPA).** Extracellular matrix structural constituents are not seen, even when the node filter is set at 12. Click here for file

Additional file 14 **Molecular Function DAG of the proteome T2-048T (UREA).** The Structural Molecule Activity (SMA) of the urea proteome contains 20 extracellular matrix structural constituents, none of which is observed in the RIPA buffer fraction DAG of Additional File 13 shown above. Thus, extracellular matrix proteins are soluble primarily in urea buffer. Click here for file

Additional file 15 **Molecular Function DAG of the matched normal proteome in RIPA buffer T2-048N (RIPA)**. Extracellular matrix structural constituents are not seen, even when the node filter is set at 17. Click here for file

Additional file 16 **Molecular Function DAG for the proteome T2-048N (UREA).** The Structural Molecule Activity of the urea proteome contains 13 extracellular matrix structural constituents. None is observed in the RIPA buffer fraction DAG of Additional File 15 shown above. Thus, extracellular matrix proteins appear to be soluble primarily in urea buffer. Click here for file

Additional file 17 **Molecular Function DAG of the matched normal proteome T2-029T (RIPA).** Extracellular matrix structural constituents are not observed, even at a node filter setting of 19. Thus, extracellular matrix proteins do not appear to be soluble in RIPA buffer. Click here for file

Additional file 18 **Molecular Function DAG for the proteome T2-029T (UREA).** The Structural Molecule Activity of the urea proteome contains 25 extracellular matrix structural constituents. None of these constituents is observed in the RIPA buffer fraction DAG of Additional File 17 shown above. Thus, extracellular matrix proteins are soluble primarily in urea buffer. Click here for file

## References

[B1] Rabilloud T (1996). Solubilization of proteins for electrophoretic analyses. Electrophoresis.

[B2] Harlow E, Lane D (1988). Antibodies: A Laboratory Manual.

[B3] Molloy MP, Herbert BR, Walsh BJ, Tyler MI, Traini M, Sanchez J-C, Hochstrasser DF, Williams KL, Gooley AA (1998). Extraction of membrane proteins by differential solubilization for separation using two-dimensional gel electrophoresis. Electrophoresis.

[B4] Rabilloud T, Adessi C, Giraudel A, Lunardi J (1997). Improvement of the solubilization of proteins in two-dimensional electrophoresis with immobilized pH gradients. Electrophoresis.

[B5] Poirier F, Lawrence D, Vigier P, Jullien P (1982). A ts T mutant of Schmidt Ruppin strain of Rous sarcoma virus restricted at 39.5 Deg C for the morphological transformation and the tumorigenicity of chicken embryo fibroblasts. Int J Cancer.

[B6] Abcam Western blotting – a beginner's guide. http://www.abcamcom/technical.

[B7] Singh S, Powell DW, Rane MJ, Millard TH, Trent JO, Pierce WM, Klein JB, Machesky LM, McLeish KR (2003). Identification of the p16-arc subunit of the arp 2/3 complex as a substrate of MAPK-activated protein kinase 2 by proteomic analysis. J Biol Chem.

[B8] Wang F, Gao J (1998). Relationship between extracellular matrix and progressive growth of malignant tumor. Zhonghua Zhongliu Zazhi.

[B9] Veeck J, Chorovicer M, Naami A, Breuer E, Zafrakas M, Bektas N, Durst M, Kristiansen G, Wild P, Hartmann A (2007). The extracellular matrix protein ITIH5 is a novel prognostic marker in invasive node-negative breast cancer and its aberrant expression is caused by promoter hypermethylation. Oncogene.

[B10] Scott GK (1997). Proteinases and proteinase inhibitors in tumor cell growth and metastasis. Cancer Journal.

[B11] Schmidt R, Bültmann A, Ungerer M, Joghetaei N, Bülbül Ö, Thieme S, Chavakis T, Toole BP, Gawaz M, Schömig A, May AE (2006). Extracellular matrix metalloproteinase inducer regulates matrix metalloproteinase activity in cardiovascular cells implications in acute myocardial infarction. Circulation.

[B12] Ruoslahti E (1992). The Walter Herbert Lecture. Control of cell motility and tumour invasion by extracellular matrix interactions. Brit J Cancer.

[B13] Ortega-Velazquez R, Gonzalez-Rubio M, Ruiz-Torres MP, Diez-Marques ML, Iglesias MC, Rodríguez-Puyol M, D R-P (2004). Collagen I upregulates extracellular matrix gene expression and secretion of TGF-beta1 by cultured human mesangial cells. Am J Physiol Cell Physiol.

[B14] Obrist P, Spizzo G, Ensinger C, Fong D, Brunhuber T, Schäfer G, Varga M, Margreiter R, Amberger A, Gastl G, Christiansen M (2004). Aberrant tetranectin expression in human breast carcinomas as a predictor of survival. J Clin Pathol.

[B15] Midwood KS, Schwarzbauer JE (2002). Tenascin-C modulates matrix contraction via focal adhesion kinase – and Rho-mediated signaling pathways. Mol Biol Cell.

[B16] Mercurius KO, Morla AO (2001). Cell adhesion and signaling on the fibronectin 1st type III repeat; requisite roles for cell surface proteoglycans and integrins. BMC Cell Biology.

[B17] Matrisian LM, Wright J, Newell K, Witty JP (1994). Matrix-degrading metalloproteinases in tumor progression. Princess Takamatsu Symp.

[B18] Macura-Biegun A (1998). The role of extracellular matrix in tumor progression. Central-Eur J Immunol.

[B19] Kelly T, Yan Y, Osborne RL, Athota AB, Rozypal TL, Colclasure JC, Chu WS (1998). Proteolysis of extracellular matrix by Invadopodia facilitates human breast cancer cell invasion and is mediated by matrix metalloproteinases. Clin Experimental Metastasis.

[B20] Klees RF, Salasznyk RM, Vandenberg S, Bennett K, Plopper GE (2007). Laminin-5 activates extracellular matrix production and osteogenic gene focusing in human mesenchymal stem cells. Matrix Biol.

[B21] Kobayashi H (1996). Mechanism of tumor cell-induced extracellular matrix degradation: inhibition of cell-surface proteolytic activity might have a therapeutic effect on tumor cell invasion and metastasis. Nippon Sanka Fujinka Gakkai Zasshi.

[B22] Hornebeck W, Maquart FX (2003). Proteolyzed matrix as a template for the regulation of tumor progression. Biomedicine & Pharmacotherapy.

[B23] Fontana S, Pucci-Minafra I, Becchi M, Freyria A-M, Minafra S (2004). Effect of collagen substrates on proteomic modulation of breast cancer cells. Proteomics.

[B24] Fata JE, Werb Z, Bissell MJ (2004). Regulation of mammary gland morphogenesis by the extracellular matrix and its remodeling enzymes. Breast Cancer Res.

[B25] Benvenuti S, Cramer R, Quinn CC, Bruce J, Zvelebil M, Corless S, Bond J, Yang A, Hockfield S, Burlingame AL (2002). Differential proteome analysis of replicative senescence in rat embryo fibroblasts. Mol Cellular Proteomics.

[B26] Espan L, Martin B, Aragues R, Chiva C, Oliva B, Andreu D, Sierra A (2005). Bcl-xL-mediated changes in metabolic pathways of breast cancer cells. Am J Pathol.

[B27] Jung SY, Malovannaya A, Wei J, O'Malley BW, Qin J (2005). Proteomic analysis of steady-state nuclear hormone receptor coactivator complexes. Molecular Endocrinol.

[B28] Pucci-Minafra I, Cancemi P, Marabeti MR, Albanese NN, Di Cara G, Taormina P, Marrazzo A (2007). Proteomic profiling of 13 paired ductal infiltrating breast carcinomas and non-tumoral adjacent counterparts. Proteomics: Clinical Applications.

[B29] Rowell C, Carpenter DM, Lamartiniere CA (2005). Chemoprevention of breast cancer, proteomic discovery of genistein action in the rat mammary gland. J Nutr.

[B30] Rucci N, Recchia I, Angelucci A, Alamanou M, Fattore AD, Fortunati D, Susa M, Fabbro D, Bologna M, Teti A (2006). Inhibition of protein kinase c-Src reduces the incidence of breast cancer metastases and increases survival in mice: implications for therapy. J Pharmacol Exp Therapeutics (JPET).

[B31] Grover A, Adamson ED (1985). Roles of extracellular matrix components in differentiating teratocarcinoma cells. J Biol Chem.

[B32] Fuchshofer R, Birke M, Welge-Lussen U, Kook D, Lütjen-Drecoll E (2005). Transforming growth factor-beta2 modulated extracellular matrix component expression in cultured human optic nerve head astrocytes. Invest Ophthalmol Vis Sci.

[B33] Conesa A, Gotz S, Garcia-Gomez JM, Terol J, Talon M, Robles M (2005). Blast2GO: a universal tool for annotation, visualization and analysis in functional genomics research. Bioinformatics.

[B34] Altschul S, Madden T, Schaffer A, Zhang J, Zhang Z, Miller W, Lipman D (1997). Gapped BLAST and PSI-BLAST: a new generation of protein database search programs. Nucleic Acids Res.

[B35] Altschul S, Gish W, Miller W, Myers E, Lipman D (1990). Basic local alignment search tool. J Mol Biol.

[B36] McEntyre J, Ostell J, (Eds) (2005). The NCBI handbook.

[B37] Ashburner M, Ball CA, Blake JA, Botstein D, Butler H, Cherry JM, Davis AP, Dolinski K, Dwight SS, Eppig JT, Harris MA, Hill DP, Issel-Tarver L, Kasarskis A, Lewis S, Matese JC, Richardson JE, Ringwald M, Rubin GM, Sherlock G (2000). Gene Ontology: tool for the unification of biology. Nature Genetics.

[B38] Ashburner M, Lewis S (2002). On ontologies for biologists: The Gene Ontology – untangling the web. Novartis Found Symp.

[B39] Davis GD, Elisee C, Newham DM, Harrison RG (1999). New fusion protein systems designed to give soluble expression in *Escherichia coli*. Biotechnol Bioeng.

[B40] Christendat D, Yee A, Dharamsi A, Kluger Y, Savchenko A, Cort JR, Booth V, Mackereth CD, Saridakis V, Ekiel I, Kozlov G, Maxwell KL, Wu N, McIntosh LP, Gehring K, Kennedy MA, Davidson AR, Pai EF, Gerstein M, Edwards AM, Arrowsmith CH (2000). Structural proteomics of an archaeon. Nat Struct Biol.

[B41] Bertone P, Kluger Y, Lan N, Zheng D, Christendat D, Yee A, Edwards AM, Arrowsmith CH, Montelione GT, Gerstein M (2001). SPINE: an integrated tracking database and data mining approach for identifying feasible targets in high-throughput structural proteomics. Nucleic Acids Res.

[B42] Ignatoski KMW, Verderame MF (1996). Lysis buffer composition dramatically affects extraction of phosphotyrosine-containing proteins. BioTechniques.

[B43] Berkelman T, Brubacher MG, Chang H (2004). Important factors influencing protein solubility for 2-D electrophoresis. BioRadiations.

[B44] Wang YP, Wang XC, Tian Q, Yang Y, Zhang Q, Zhang JY, Zhang YC, Wang ZF, Wang Q, Li H, Wang JZ (2006). Endogenous overproduction of β-amyloid induces tau hyperphosphorylation and decreases the solubility of tau in N2a cells. Journal of Neural Transmission.

[B45] Baan B, Dam Hv, Zon GCM van der, Maassen JA, Ouwens DM (2006). The Role of c-Jun N-terminal kinase, p38, and extracellular signal-regulated kinase in insulin-induced Thr69 and Thr71 phosphorylation of activating transcription factor 2. Molecular Endocrinol.

[B46] Gao H, Wu B, Giese R, Zhu Z (2007). Xom interacts with and stimulates transcriptional activity of LEF1/TCFs: implications for ventral cell fate determination during vertebrate embryogenesis. Cell Research.

[B47] DeSeau V, Rosen N, Bolen JB (1987). Analysis of pp60c-src tyrosine kinase activity and phosphotyrosyl phosphatase activity in human colon carcinoma and normal human colon mucosal cells. J Cellular Biochem.

[B48] Zapata JM, Takahashi R, Salvesen GS, Reed JC (1998). Granzyme release and caspase activation in activated human T-lymphocytes. J Biol Chem.

[B49] Granier F (1988). Extraction of plant proteins for two-dimensional electrophoresis. Electrophoresis 1988.

[B50] Ames GF-L, Nikaido K (1976). Two-dimensional gel electrophoresis of membrane proteins. Biochemistry.

[B51] O'Farrell PH (1975). High resolution two-dimensional electrophoresis of proteins. J Biol Chem.

[B52] Padgaonkar V, Giblin F, Reddy V (1989). Disulfide cross-linking of urea-insoluble proteins in rabbit lenses treated with hyperbaric oxygen. Exp Eye Res.

[B53] Harding JJ (1972). Conformational changes in human lens proteins in cataract. Biochem J.

[B54] Fleschner CR (2006). Connexin 46 and connexin 50 in Selenite cataract. Ophthalmic Research.

[B55] Weber DJ, McFadden PN (1995). A heterogenous set of urea-insoluble proteins in dividing PC12 pheochromocytoma cells is passed on to at least the generation of great-granddaughter cells. J Protein Chem.

[B56] Wu H, Gao J, Sharif WD, Davidson MK, Wahls WP (2004). Purification, folding, and characterization of Rec12 (Spo11) meiotic recombinase of fission yeast. Protein Expression Purification.

[B57] Moudjou M, Paintrand M, Vigues B, Bornens M (1991). A human centrosomal protein is immunologically related to basal body-associated proteins from lower eukaryotes and is involved in the nucleation of microtubules. J Cell Biol.

