# Supplementary figures and images for: Sample prep for proteomics of breast cancer: proteomics and gene ontology reveal dramatic differences in protein solubilization preferences of radioimmunoprecipitation assay and urea lysis buffers
Source: Proteome Sci. 2008 Oct 24;6:30. doi: 10.1186/1477-5956-6-30 (PMC2600628; doi:10.1186/1477-5956-6-30)

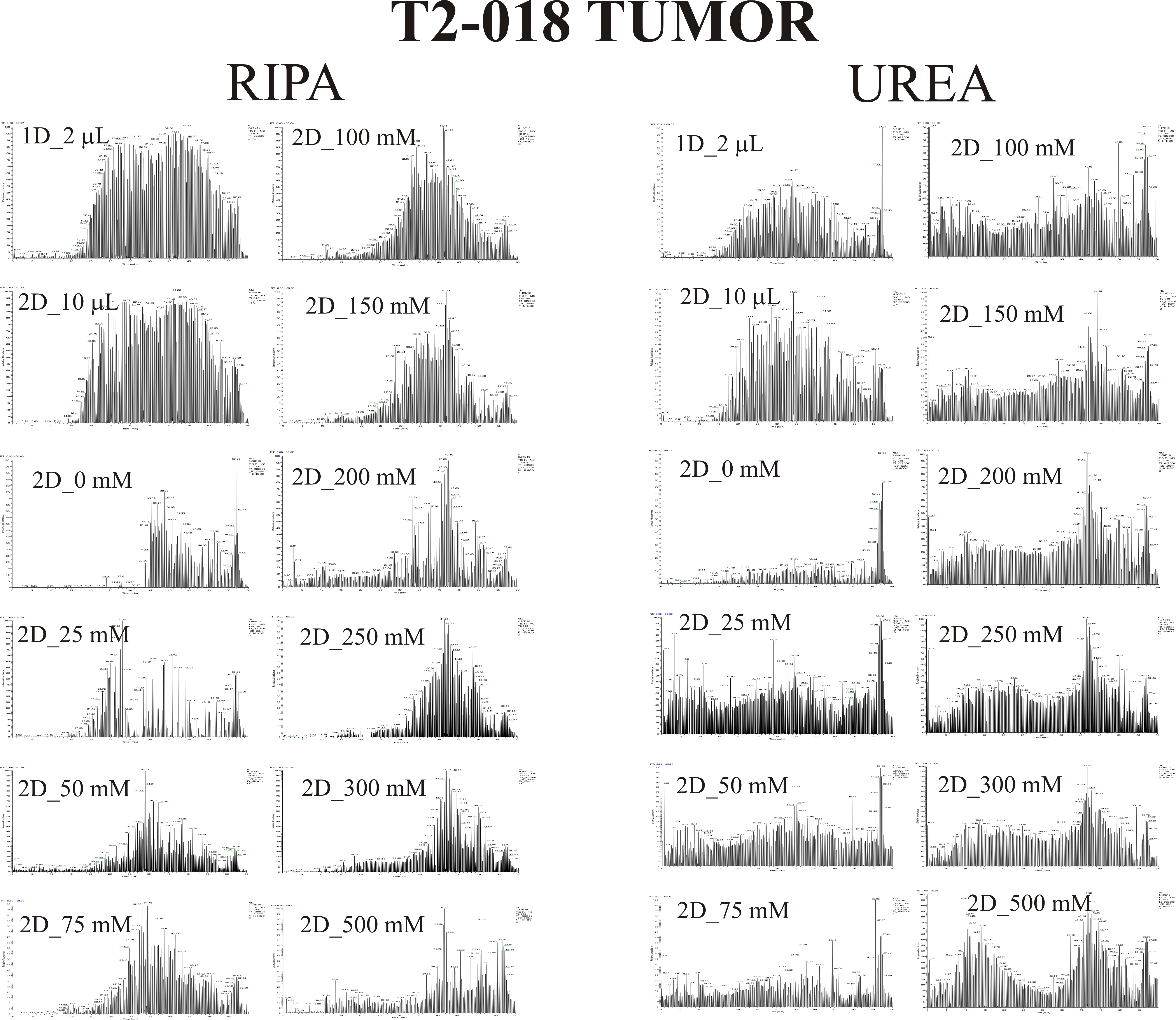

Supplement: Additional file 2 — MudPIT Mass Spectra of the breast tumor T2-018 TUMOR. The set of 12 MudPIT mass spectra of the RIPA-soluble fraction are shown at left, whereas those for the urea-soluble fraction are shown at right. A typical MudPIT experiment consists of a 12-cycle run in which a 60-minute nano-LC gradient is run for each of: 1. 1D_2 μL sample; 2. 2D_10 μL sample; 3. 2D_0 mM NH4COO-; 4. 2D_25 mM NH4COO-; 5. 2D_50 mM NH4COO-; 6. 2D_75 mM NH4COO-; 7. 2D_100 mM NH4COO-; 8. 2D_150 mM NH4COO-; 9. 2D_200 mM NH4COO-; 10. 2D_250 mM NH4COO-; 11. 2D_300 mM NH4COO-, and 12. 2D_500 mM NH4COO-. NH4COO- is ammonium formate. [file 1477-5956-6-30-S2.tiff]

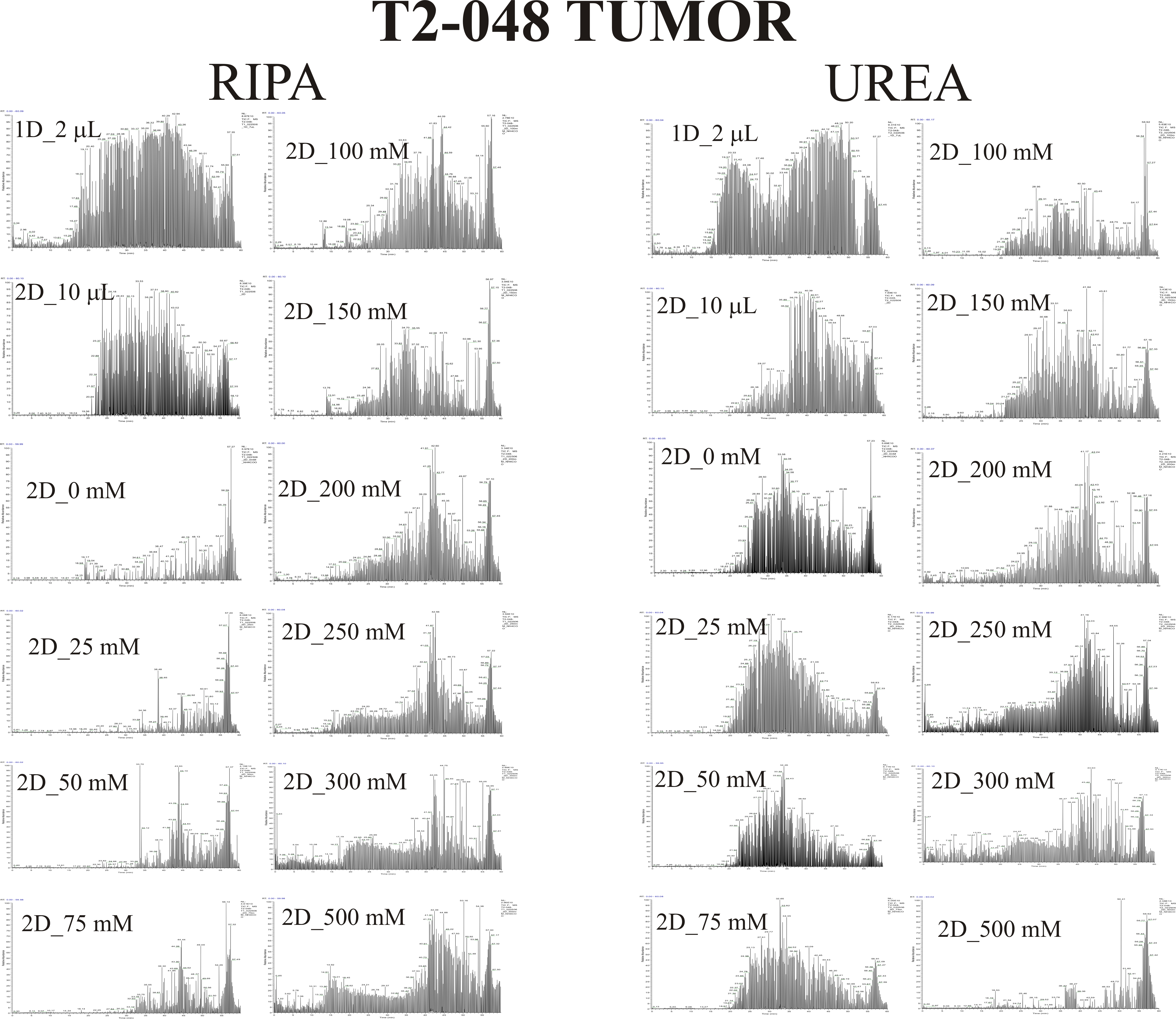

Supplement: Additional file 3 — MudPIT Mass Spectra of the breast tumor T2-048 TUMOR. MudPIT Mass Spectra of the breast tumor T2-048 TUMOR. Spectra of RIPA-soluble fraction are shown at left, whereas those for the urea-soluble fraction are shown at right. [file 1477-5956-6-30-S3.tiff]

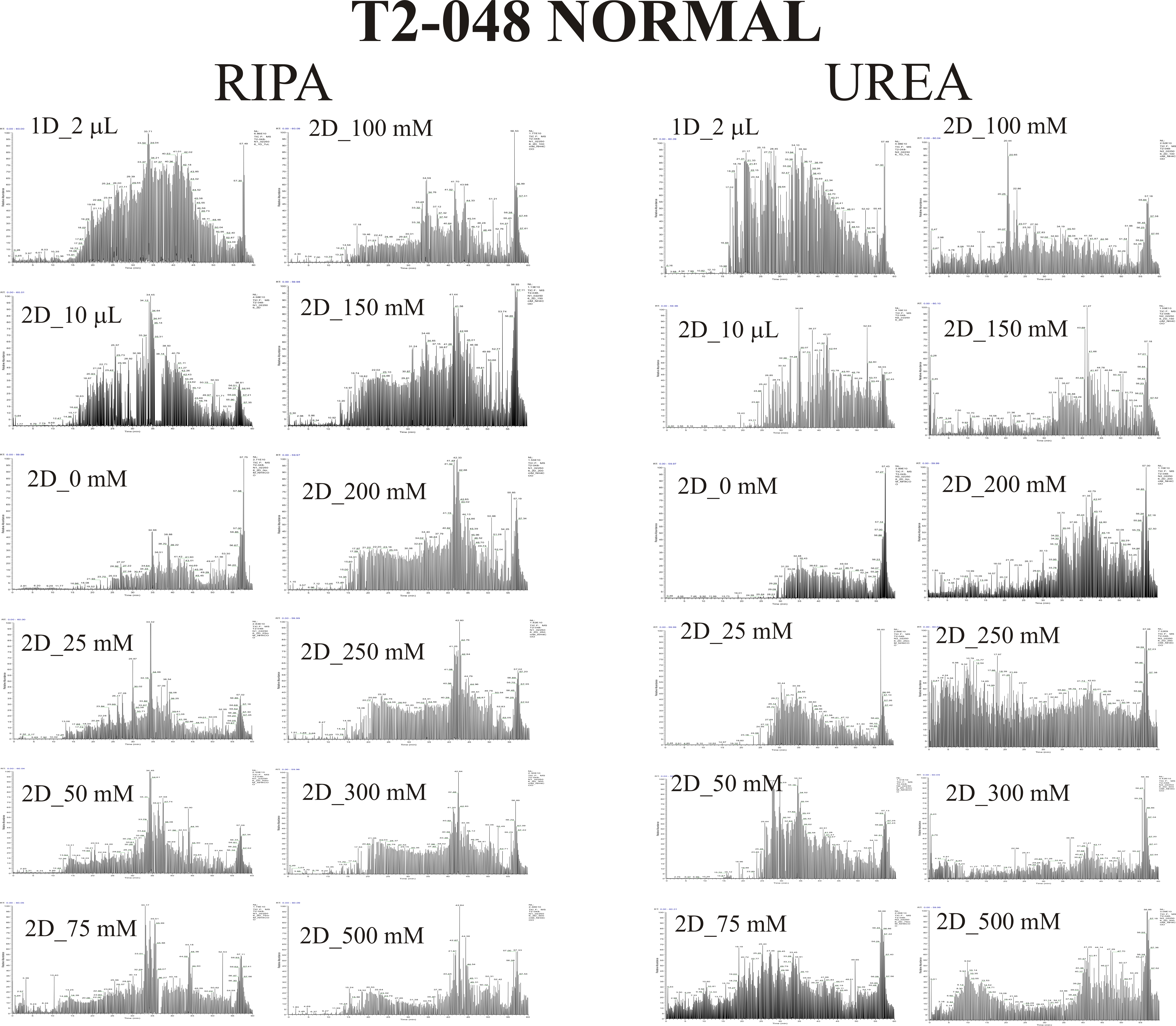

Supplement: Additional file 4 — MudPIT Mass Spectra of the matched normal breast tissue T2-048 NORMAL. The set of 12 MudPIT mass spectra of the RIPA-soluble fraction are shown at left, whereas those for the urea-soluble fraction are shown at right. [file 1477-5956-6-30-S4.tiff]

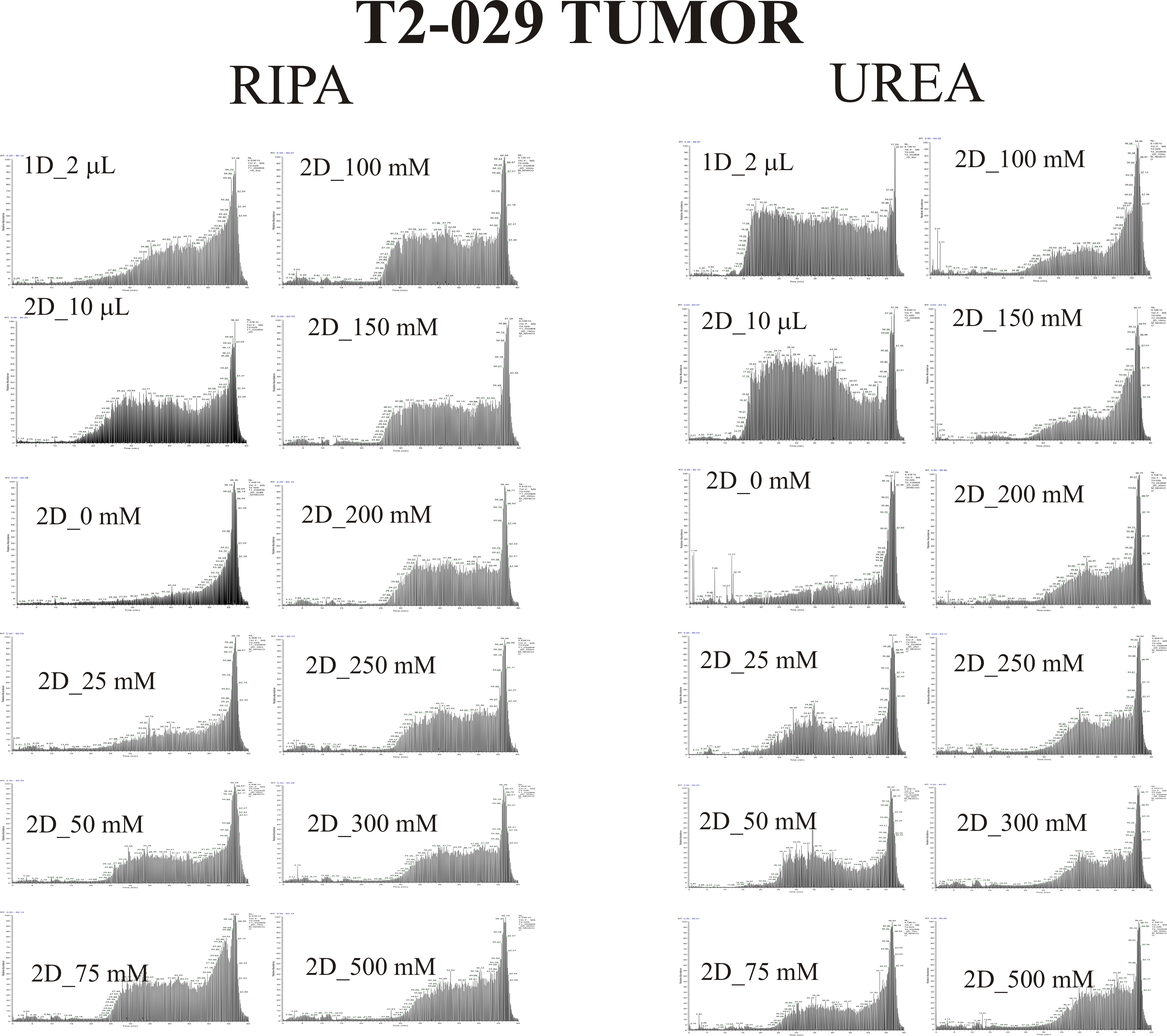

Supplement: Additional file 5 — MudPIT Mass Spectra of the bilateral breast tumor T2-029 TUMOR. The set of 12 MudPIT mass spectra of the RIPA-soluble fraction are shown at left, whereas those for the urea-soluble fraction are shown at right. [file 1477-5956-6-30-S5.tiff]

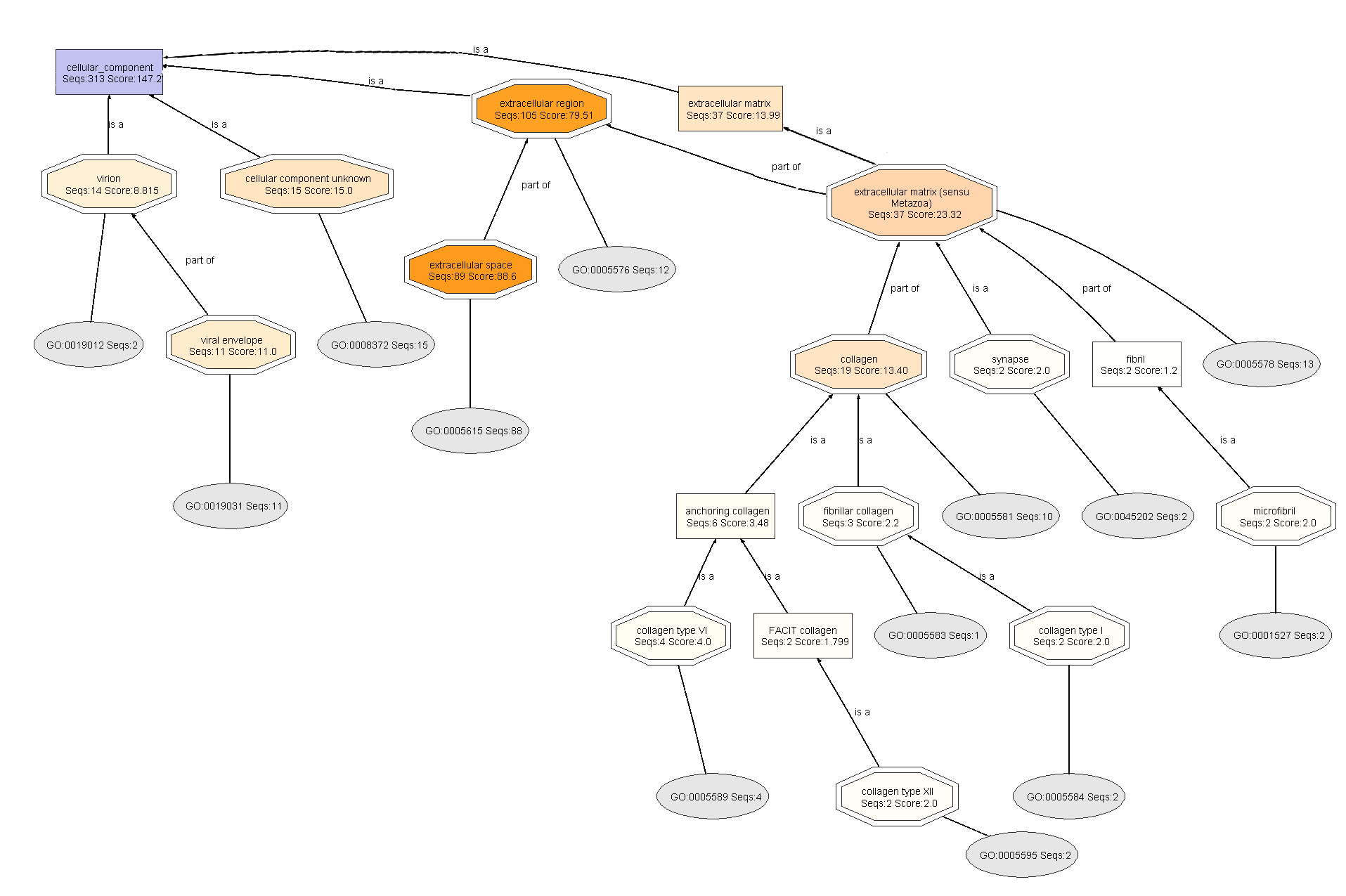

Supplement: Additional file 6 — Expanded view of the extracellular region of the Cellular Component DAG for the bilateral proteome T2-029T (UREA). The node filter was reduced to 0 to obtain this complete display. In contrast, lowering the DAG node filter for the RIPA DAG counterpart did not produce appreciable change in the number of nodes displayed within the extracellular region. [file 1477-5956-6-30-S6.png]

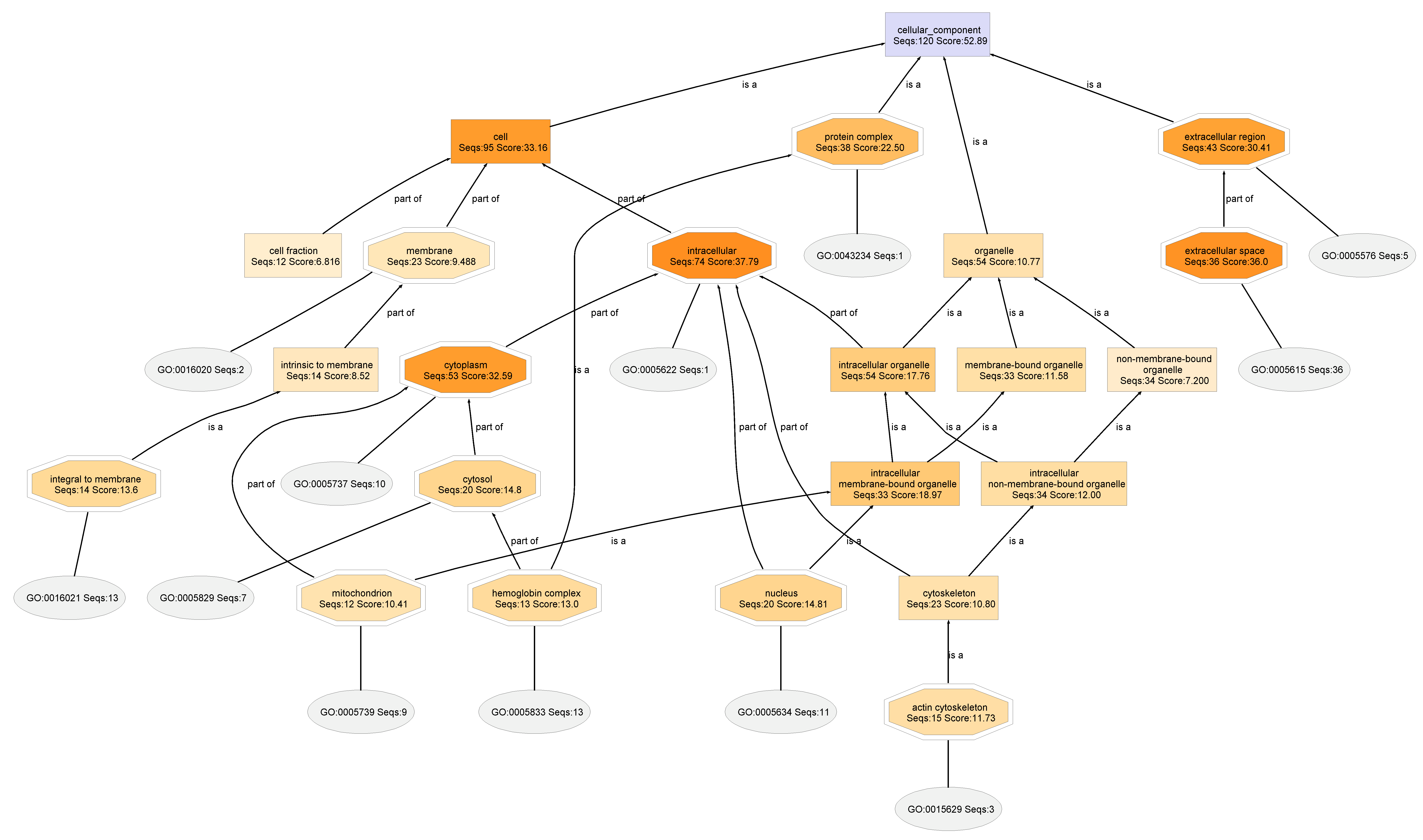

Supplement: Additional file 7 — Cellular Component DAG for the proteome T2-048T (RIPA). Extracellular matrix proteins are not seen, even when the node filter is lowered to 10. [file 1477-5956-6-30-S7.png]

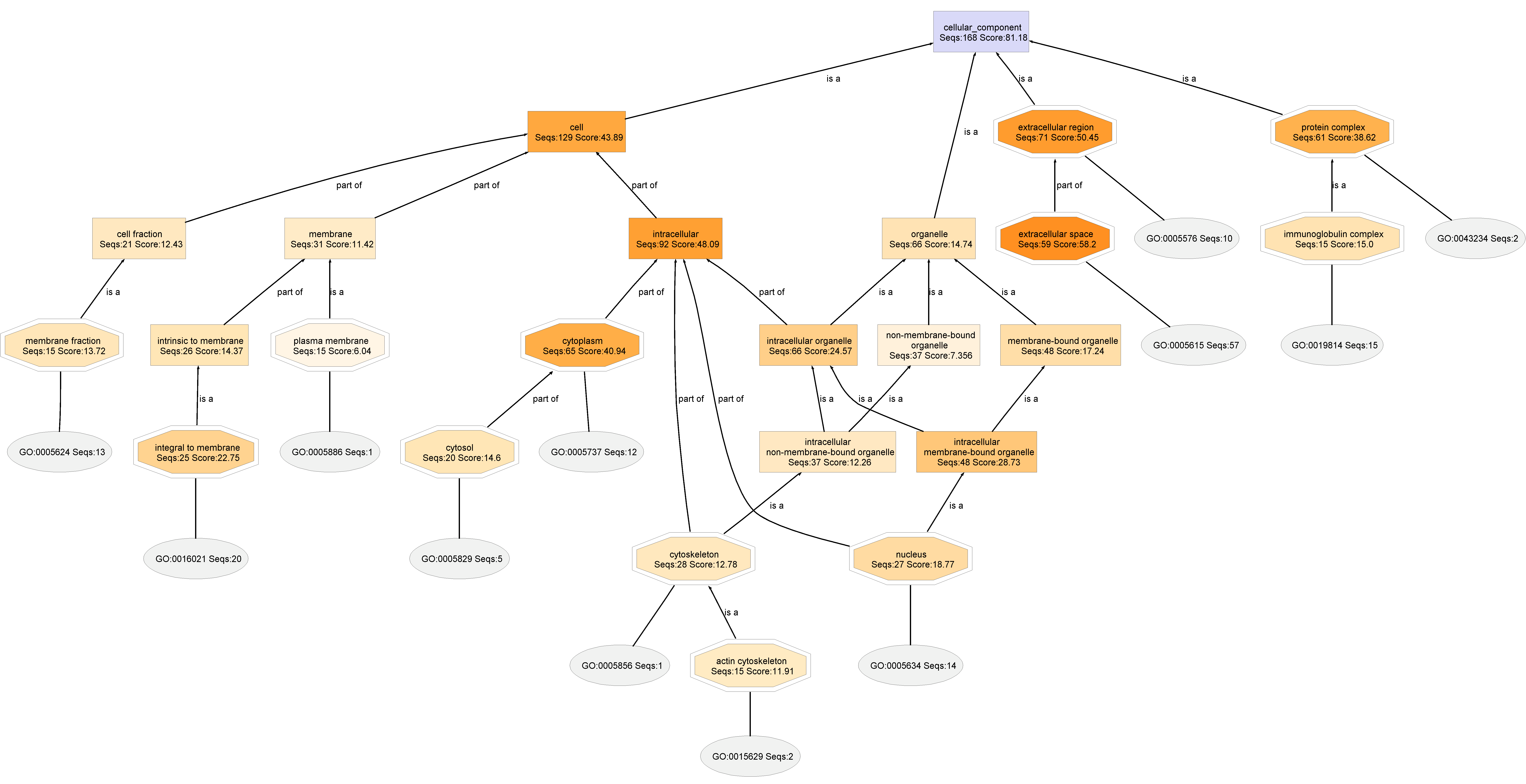

Supplement: Additional file 9 — Cellular Component DAG of the matched normal proteome T2-048N (RIPA). Extracellular matrix proteins are not seen, even at a node filter setting of 14. [file 1477-5956-6-30-S9.png]

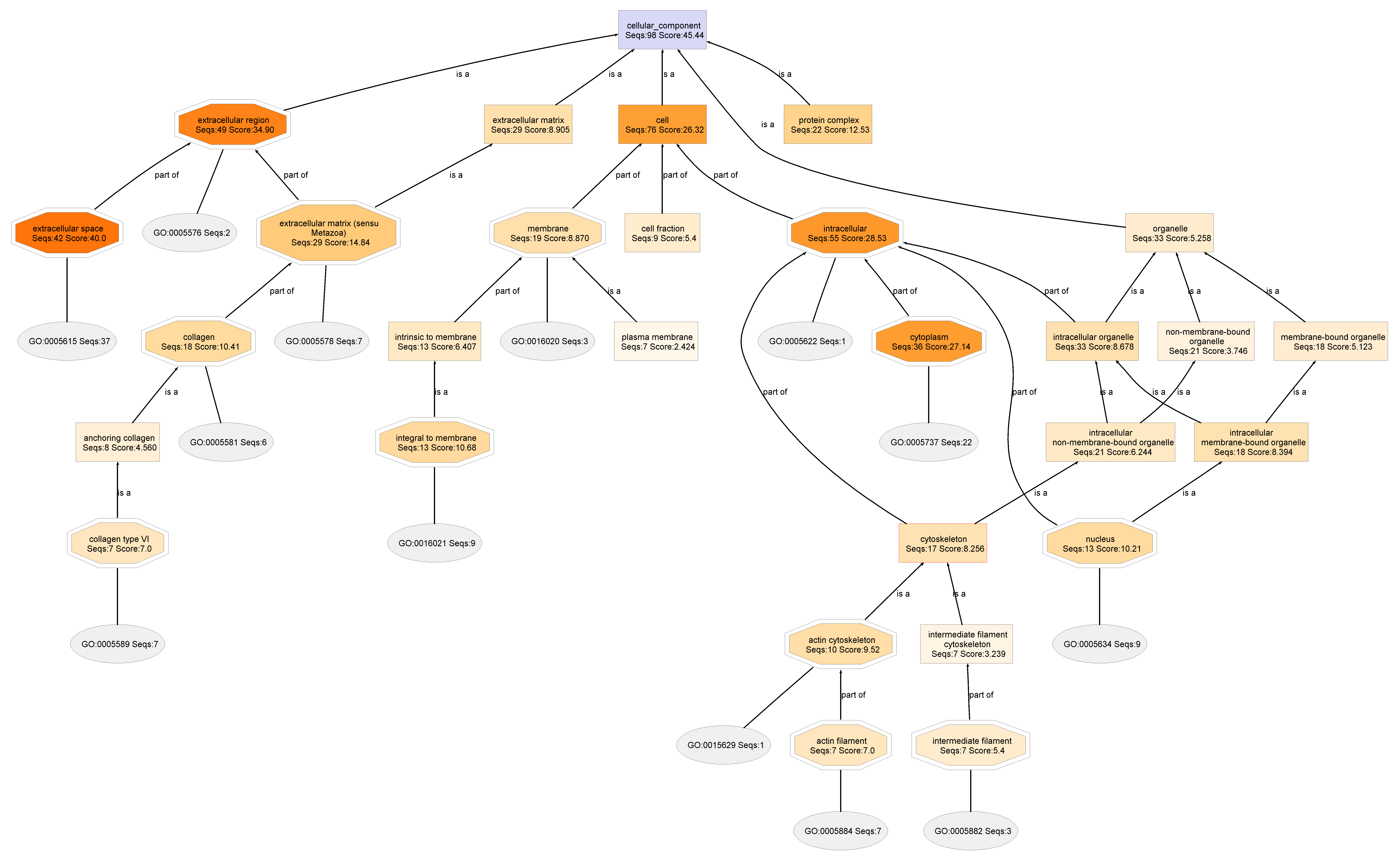

Supplement: Additional file 10 — Cellular Component DAG of the matched normal proteome T2-048N (UREA). Twenty-nine extracellular matrix proteins are present in this urea buffer fraction of T2-048N, even when the DAG is displayed with a node filter setting of just 5 (cf. Node filter is 14 in Additional File 9 above); no extracellular matrix proteins are present in the RIPA buffer fraction of this proteome, shown in Additional File 9 above. [file 1477-5956-6-30-S10.png]

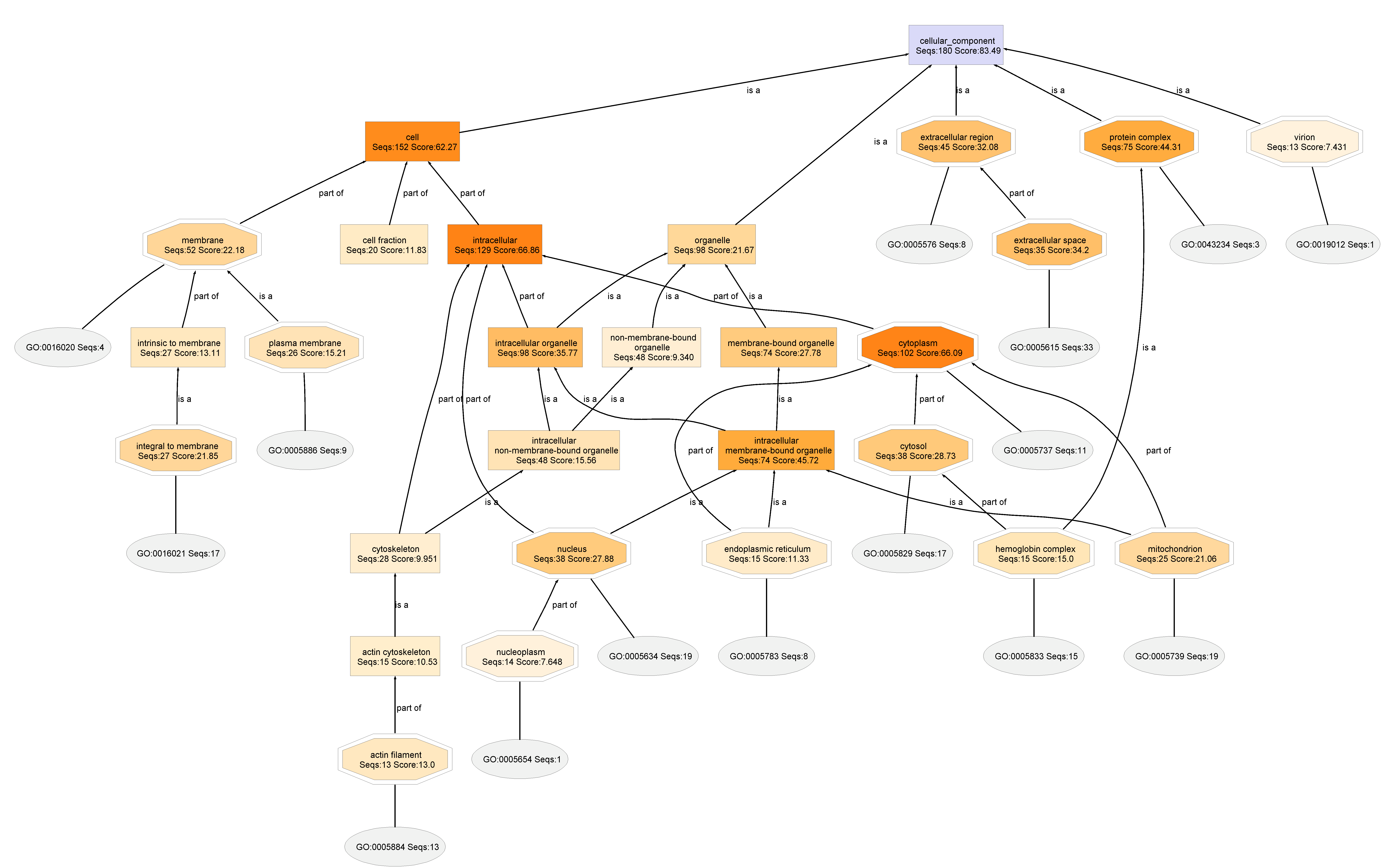

Supplement: Additional file 11 — Cellular Component DAG of the bilateral Adenocarcinoma proteome T2-029T (RIPA). Extracellular matrix proteins are not seen, even at a node filter setting of 12. [file 1477-5956-6-30-S11.png]

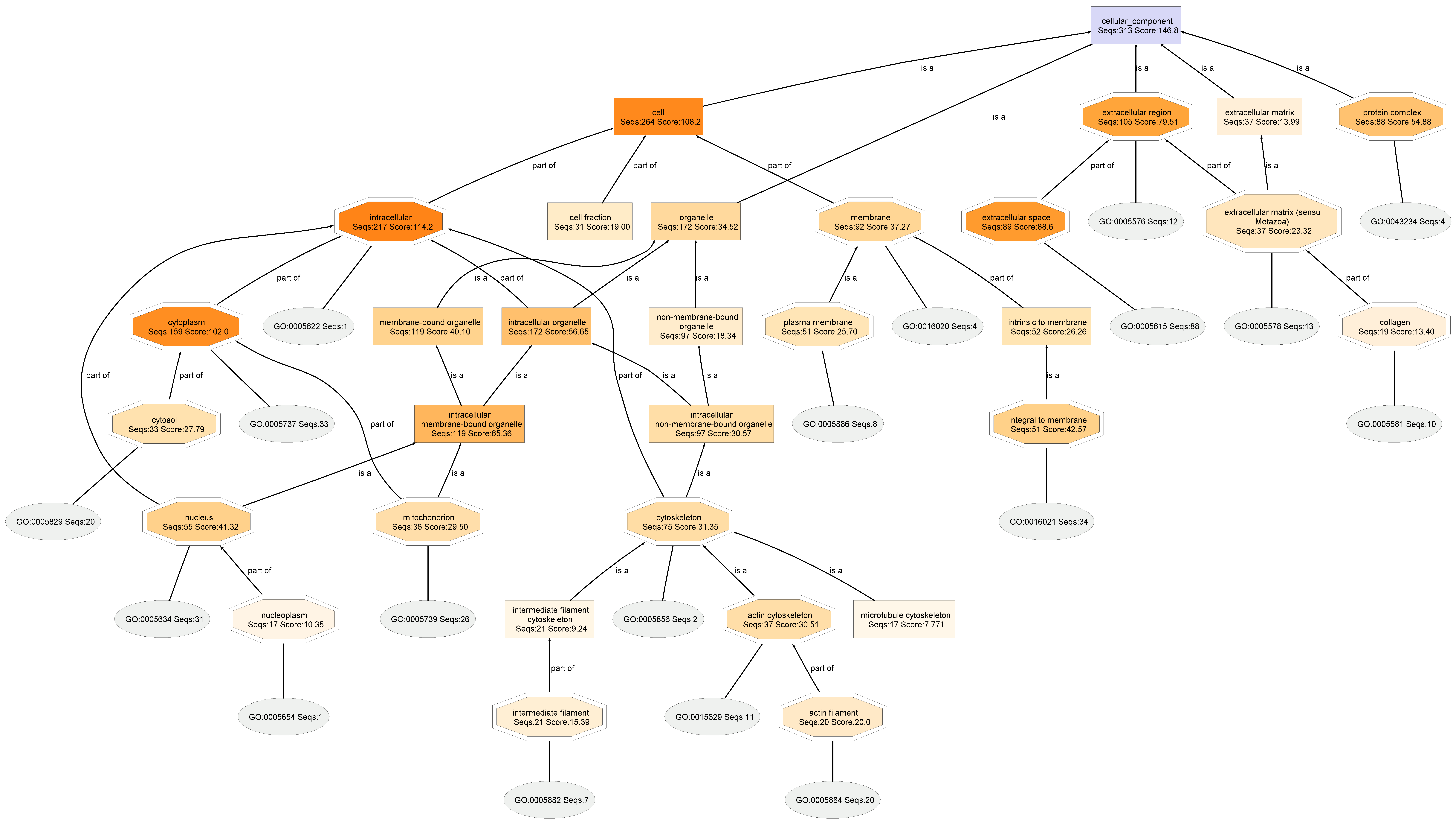

Supplement: Additional file 12 — Cellular Component DAG of the bilateral Adenocarcinoma proteome T2-029T (UREA). Thirty-seven extracellular matrix proteins are observed, at a node filter setting of 16. No extracellular matrix proteins are observed in the RIPA buffer fraction of this proteome, which is shown in Additional File 11 above. [file 1477-5956-6-30-S12.png]

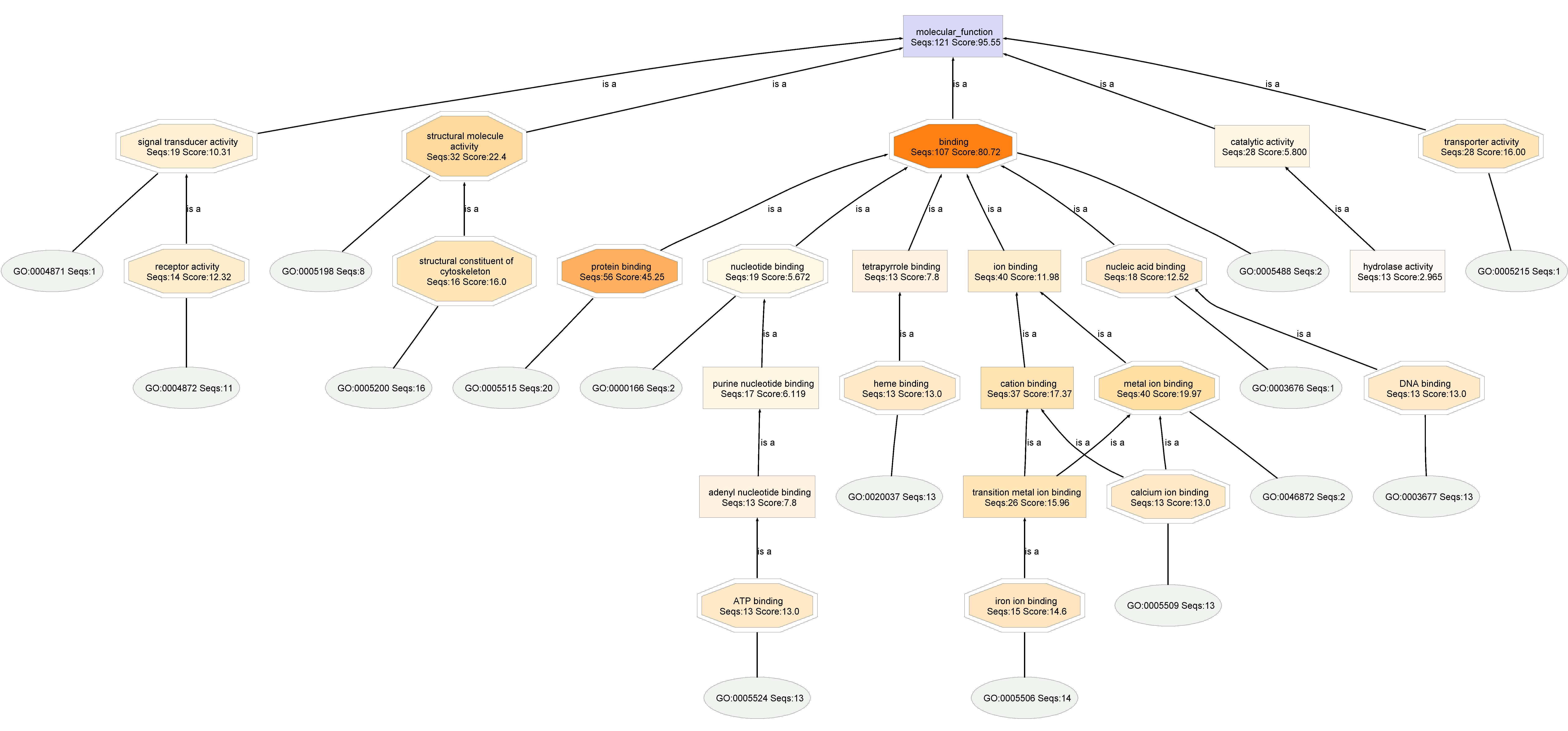

Supplement: Additional file 13 — Molecular Function DAG for the proteome T2-048T (RIPA). Extracellular matrix structural constituents are not seen, even when the node filter is set at 12. [file 1477-5956-6-30-S13.png]

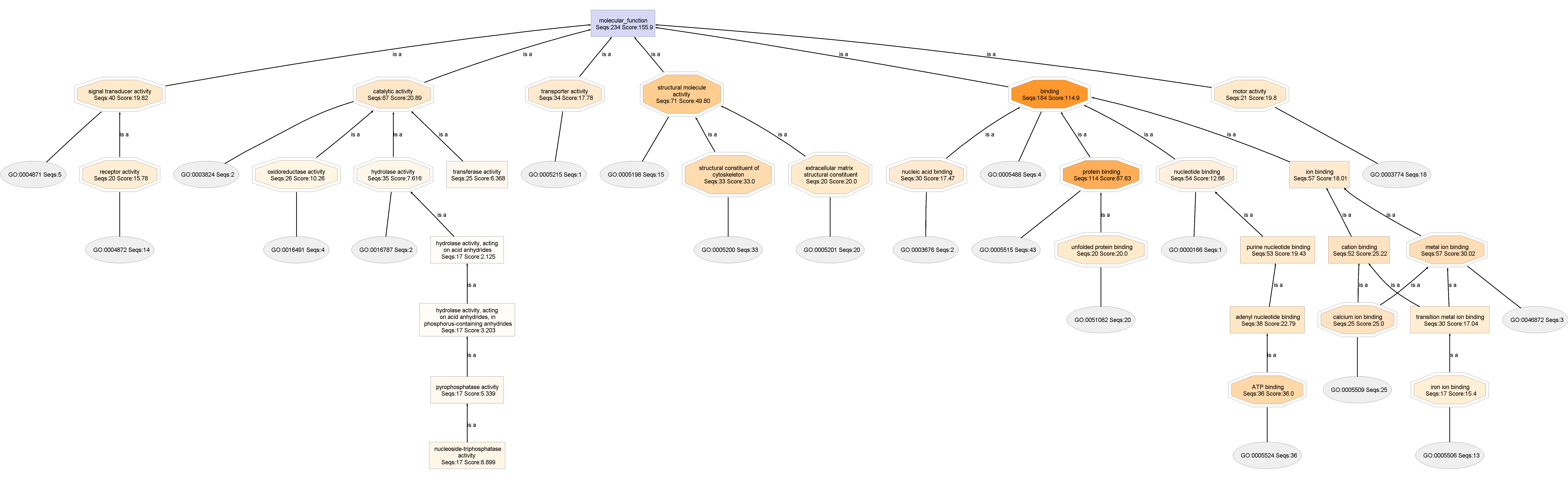

Supplement: Additional file 14 — Molecular Function DAG of the proteome T2-048T (UREA). The Structural Molecule Activity (SMA) of the urea proteome contains 20 extracellular matrix structural constituents, none of which is observed in the RIPA buffer fraction DAG of Additional File 13 shown above. Thus, extracellular matrix proteins are soluble primarily in urea buffer. [file 1477-5956-6-30-S14.png]

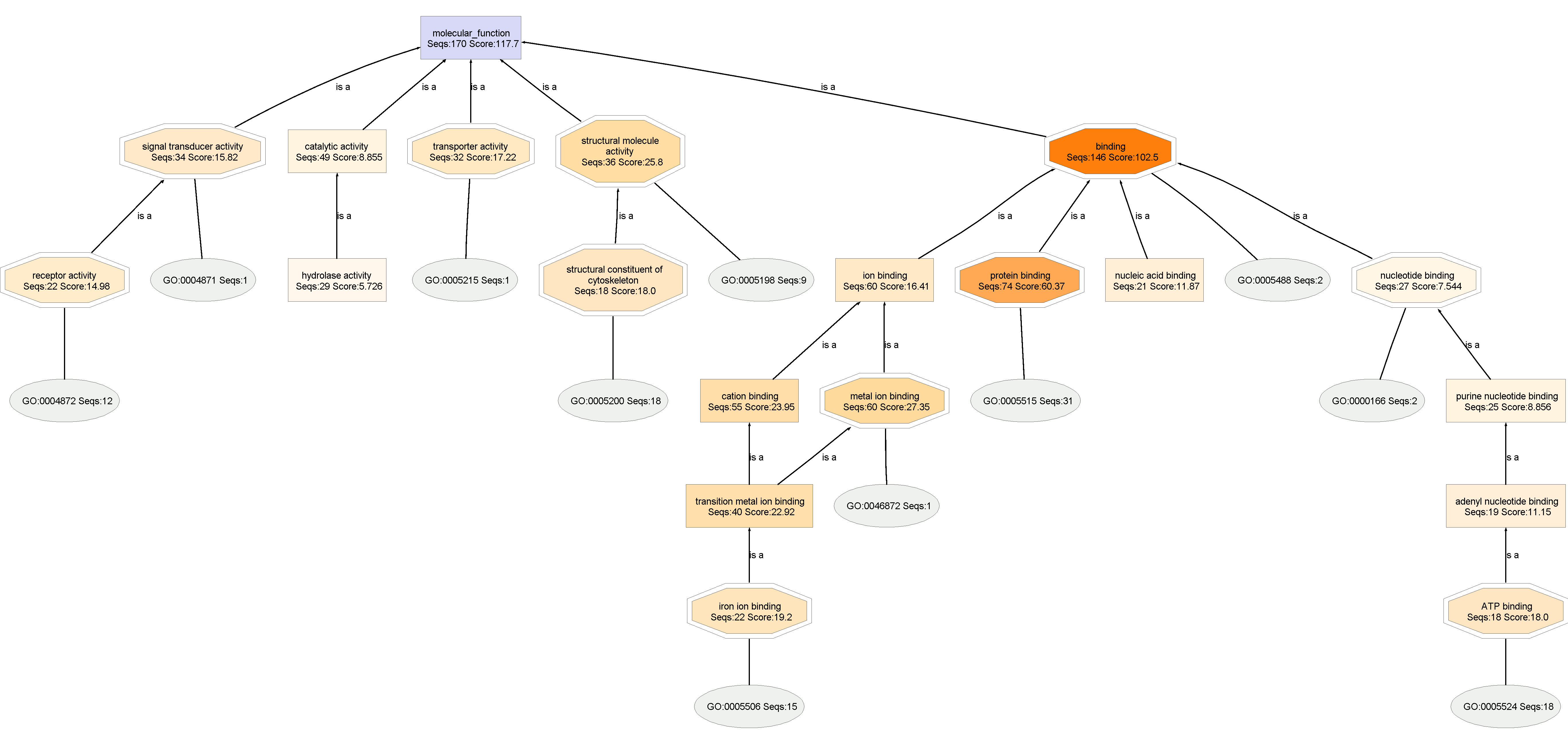

Supplement: Additional file 15 — Molecular Function DAG of the matched normal proteome in RIPA buffer T2-048N (RIPA). Extracellular matrix structural constituents are not seen, even when the node filter is set at 17. [file 1477-5956-6-30-S15.png]

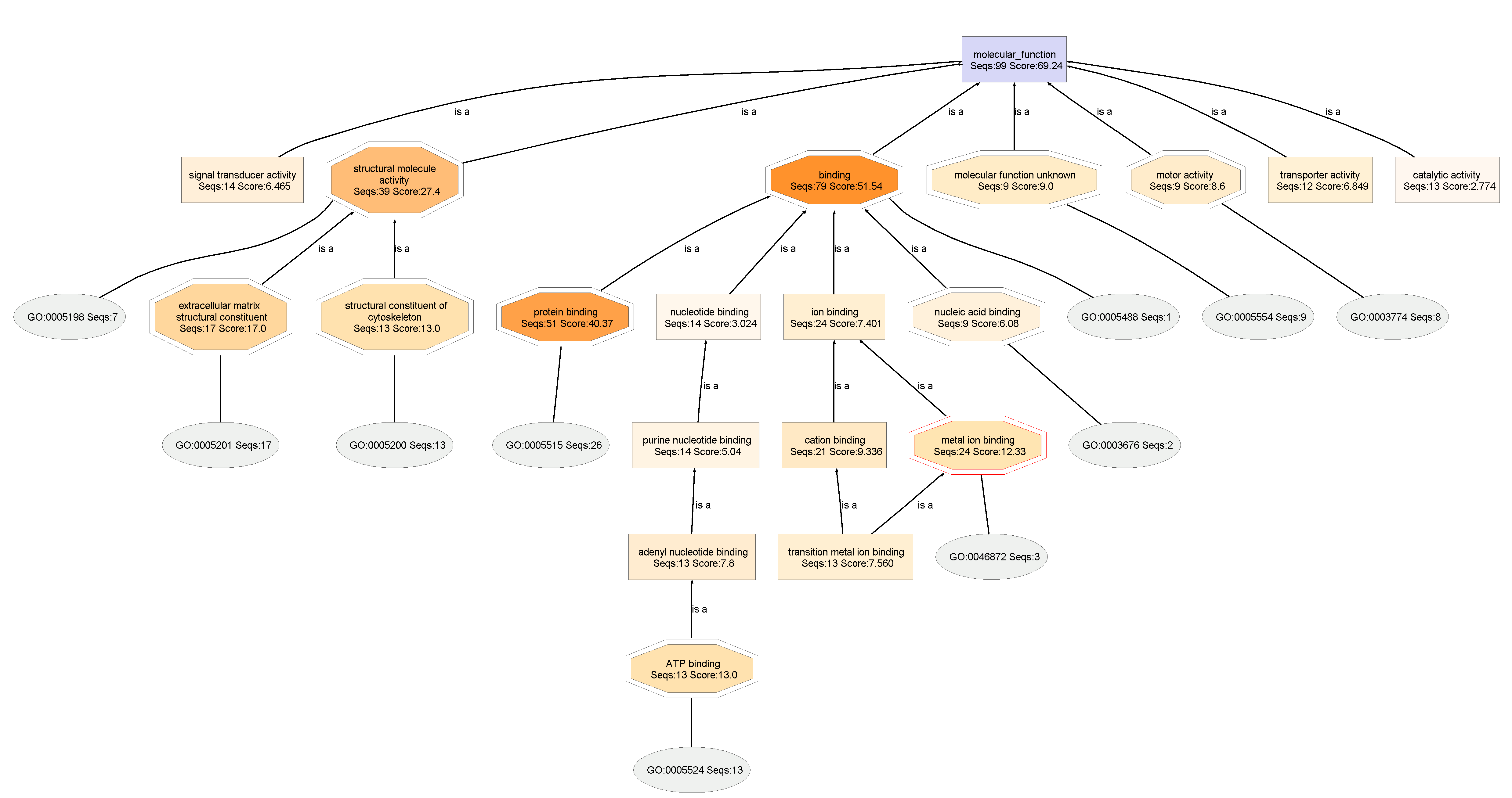

Supplement: Additional file 16 — Molecular Function DAG for the proteome T2-048N (UREA). The Structural Molecule Activity of the urea proteome contains 13 extracellular matrix structural constituents. None is observed in the RIPA buffer fraction DAG of Additional File 15 shown above. Thus, extracellular matrix proteins appear to be soluble primarily in urea buffer. [file 1477-5956-6-30-S16.png]

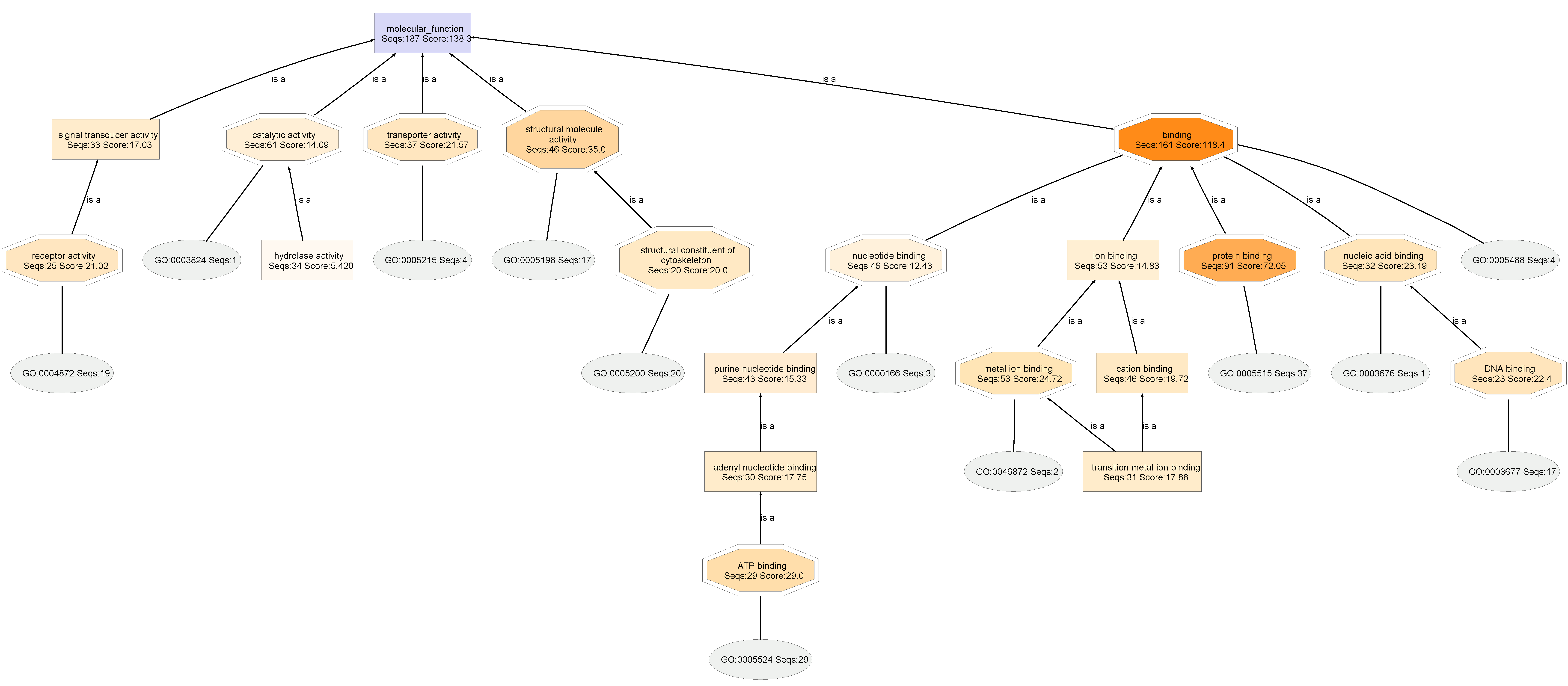

Supplement: Additional file 17 — Molecular Function DAG of the matched normal proteome T2-029T (RIPA). Extracellular matrix structural constituents are not observed, even at a node filter setting of 19. Thus, extracellular matrix proteins do not appear to be soluble in RIPA buffer. [file 1477-5956-6-30-S17.png]

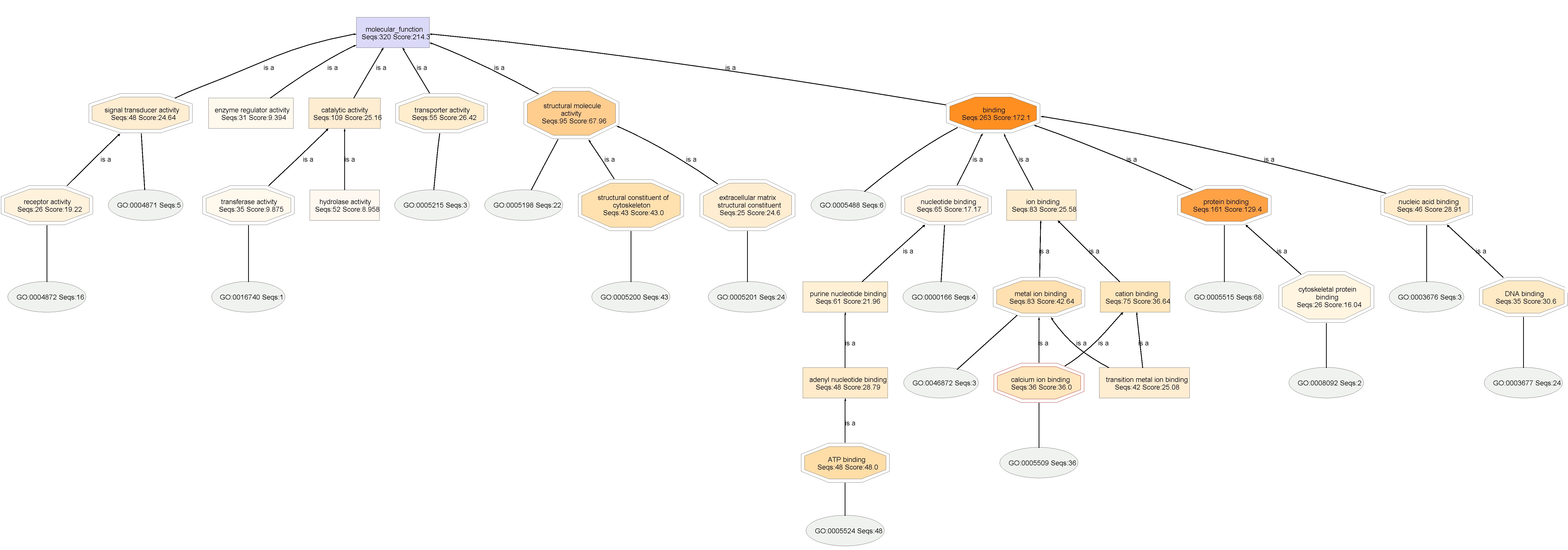

Supplement: Additional file 18 — Molecular Function DAG for the proteome T2-029T (UREA). The Structural Molecule Activity of the urea proteome contains 25 extracellular matrix structural constituents. None of these constituents is observed in the RIPA buffer fraction DAG of Additional File 17 shown above. Thus, extracellular matrix proteins are soluble primarily in urea buffer. [file 1477-5956-6-30-S18.png]
